# Titanium biogenic nanoparticles to help the growth of *Trichoderma harzianum* to be used in biological control

**DOI:** 10.1186/s12951-023-01918-y

**Published:** 2023-05-25

**Authors:** Tatiane Pasquoto-Stigliani, Mariana Guilger-Casagrande, Estefânia V. R. Campos, Tais Germano-Costa, Natalia Bilesky-José, Bianca B. Migliorini, Leandro O. Feitosa, Bruno T. Sousa, Halley C. de Oliveira, Leonardo F. Fraceto, Renata Lima

**Affiliations:** 1 Laboratory for Evaluation of the Bioactivity and Toxicology of Nanomaterials, University of Sorocaba (UNISO), Sorocaba, São Paulo Brazil; 2 Institute of Science and Technology of Sorocaba, Laboratory of Environmental Nanotechnology, State University of São Paulo (UNESP), Sorocaba, São Paulo Brazil; 3 Departament of Animal and Plant Biology, University of Londrina (UEL), Londrina, Paraná Brazil

**Keywords:** Nanoparticle biosynthesis, Green nanoparticle route, Biological control, Metal oxide nanoparticles, *Sclerotinia sclerotiorum*, Phytopathogen

## Abstract

**Background:**

The biogenic synthesis of metallic nanoparticles is a green alternative that reduces the toxicity of this nanomaterials and may enable a synergy between the metallic core and the biomolecules employed in the process enhancing biological activity. The aim of this study was to synthesize biogenic titanium nanoparticles using the filtrate of the fungus *Trichoderma harzianum* as a stabilizing agent, to obtain a potential biological activity against phytopathogens and mainly stimulate the growth of *T. harzianum*, enhancing its efficacy for biological control.

**Results:**

The synthesis was successful and reproductive structures remained in the suspension, showing faster and larger mycelial growth compared to commercial *T. harzianum* and filtrate. The nanoparticles with residual *T. harzianum* growth showed inhibitory potential against *Sclerotinia sclerotiorum* mycelial growth and the formation of new resistant structures. A great chitinolytic activity of the nanoparticles was observed in comparison with *T. harzianum*. In regard to toxicity evaluation, an absence of cytotoxicity and a protective effect of the nanoparticles was observed through MTT and Trypan blue assay. No genotoxicity was observed on V79-4 and 3T3 cell lines while HaCat showed higher sensitivity. Microorganisms of agricultural importance were not affected by the exposure to the nanoparticles, however a decrease in the number of nitrogen cycling bacteria was observed. In regard to phytotoxicity, the nanoparticles did not cause morphological and biochemical changes on soybean plants.

**Conclusion:**

The production of biogenic nanoparticles was an essential factor in stimulating or maintaining structures that are important for biological control, showing that this may be an essential strategy to stimulate the growth of biocontrol organisms to promote more sustainable agriculture.

**Graphical Abstract:**

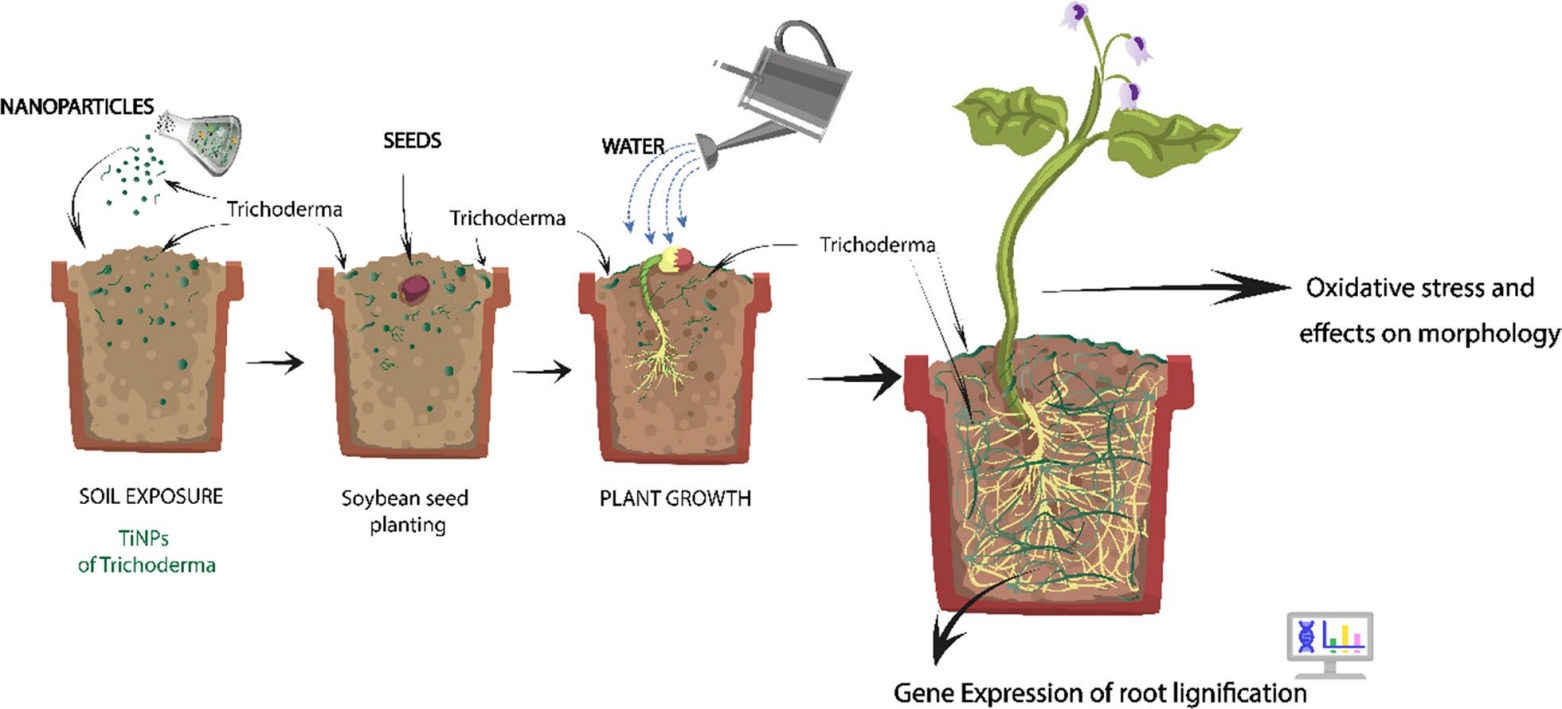

## Introduction

In recent years, pest biological control has emerged, aiming mainly at developing more sustainable crops and reducing the use of hazardous pesticides. However, this journey faces obstacles to total success, such as the viability of the microorganisms used as biocontrol agents, the product storage and the interference of combined products. There are still some challenges to using microorganisms as the primary way to control agricultural pathogens in large crops.

According to Liu et al. the application of nanotechnology in agriculture has positive aspects [1]. Still, there are some gaps to be addressed, mainly regarding the transference of the knowledge from the laboratory bench to the application of the products in the field. In this context, micro and nanotechnology need more studies to enhance the biological activity of microorganisms since these technologies enable applications aiming at both soil fertilization and pest control [1–5]. A review about nanoparticles as nanofertilizers by Fatima et al. showed that there is an incentive for the use of biological organisms to develop a sustainable agrosystem in the future. It is believed that this goal will be achieved with the use of nanofertilizers which will come to replace biofertilizers and synthetic fertilizers [3]. To guarantee security and sustainable development, some nanoplatforms were developed with several requirements to ensure agricultural revolution [6].

A wide range of possibilities highlights nanotechnology to be applied in the agricultural sector as different strategies may be used, such as nanosensors [7, 8], polymeric nanocapsules for the controlled release of pesticides [9–12], metallic nanoparticles [13, 14] and metal oxide nanoparticles such as titanium dioxide which did not show phytotoxicity and may promote plant growth [15–17]. Concerning microtechnology, there is the possibility of encapsulating organisms to ensure field resistance [4].

The biogenic synthesis of metallic nanoparticles employing living organisms and their metabolites is considered a sustainable alternative that may enable the synergy between the metallic core and the metabolites from the organism, which remain around the metallic core forming a capping. This capping may contain enzymes acting as pest control agents and as direct or indirect enhancers for soil fertilization [18–22].

*Trichoderma* spp is widely used in biological control due to its specificity. This fungus controls the development of several phytopathogens and stimulates plant growth [23–27]. *Sclerotinia sclerotiorum* (White mold) is one of the phytopathogenic fungi susceptible to *Trichoderma* spp biological activity. This pest affects the development of crops of economic importance, such as soybean, beans, potato and tomato, causing losses of up to 100% of the yield [28, 29]. *Trichoderma spp.* is responsible for controlling phytopathogens through the combined action of different routes such as competition, the release of metabolites in plants' rhizosphere and systemic resistance [30, 31]. Regarding the use of Trichoderma as a fertilizer, studies show that in addition to the activity against phytopathogens, Trichoderma also promotes plant growth, as it induces the production of phytohormones that activate plant supplements and produce secondary metabolites, being used as biofertilizers [32–35]. According to Marra et al., Trichoderma spp. will significantly contribute to developing a new generation of environmentally friendly biostimulants and bioprotectors in the coming years [36].

The present study brings biogenic titanium nanoparticles that were designed to stimulate the growth of Trichoderma spp, aiming at better product efficiency, increase in shelf life, improvement of biocontrol activity and enhancement of Trichoderma colonization in the field. A previous study by our group showed that biogenic iron oxide nanoparticles promoted the stimulation of Trichoderma growth due to the presence of germinative structures remaining from the synthesis, apparently being a way to ensure more significant development and maintenance of the fungus in the field [22]. In this way, it was decided to synthesize titanium nanoparticles (titanium oxide IV/Rutile) since studies showed the absence of toxicity and its biocompatible properties [37]. Especially in the agricultural sector, the use of titanium dioxide showed a stimulating effect triggering the increase of plant biomass production and photosynthetic rate [38, 39], stimulation of seed germination, higher nutrient absorption rate by the roots, stimulation of enzymatic activity, resistance to stress and, consequently, a higher yield [16]. Another deciding factor was the finding that the cultivation of Trichoderma in the presence of *S. sclerotiorum* cell wall stimulates the production of chitinases, β-glucanases and proteases [40, 41]. This finding triggered the biogenic synthesis of titanium nanoparticles using *Trichoderma harzianum* with and without enzymatic stimulation by the phytopathogen *S. sclerotiorum*.

Accordingly, the objective of this study was to synthesize biogenic titanium nanoparticles using titanium oxide IV (Rutile) as a metallic precursor and the filtrate of the fungus *Trichoderma harzianum* (with and without enzymatic stimulation by the cell wall of *Sclerotinia sclerotiorum*) as a stabilizing agent, in an attempt to obtain not only nanoparticles with biological activity against phytopathogens but mainly nanoparticles that stimulate the growth of Trichoderma, enhancing its efficacy for biological control. Growth kinetics, activity against *S. sclerotiorum* and toxicity in soil and plants were also evaluated.

## Materials and methods

### Materials

The commercial product Ecotrich WP 1 × 10^10^ UFC/g (Balagro^™^, Brazil) was used to obtain the initial culture of *T. harzianum* to synthesize the nanoparticles. Titanium IV Rutile oxide (≥ 99.98% purity), MTT (3-(4,5-dimethylthiazolyl-2)-2,5-diphenyltetrazolium bromide) salt (≥ 97.5% purity), Trypan Blue dye and Resazurin sodium salt were purchased from Sigma Aldrich Chemicals, USA. Potato dextrose agar (PDA), potato dextrose broth (PDB) and Müeller Hinton broth (MHB) were obtained from Kasvi, Brazil. Dimethylsulfoxide (DMSO) (99.9% purity) was obtained from Dinâmica, Brazil; Power Soil^™^ DNA Isolation Kit was purchased from Qiagen. Qubit^™^ RNA HS Assay Kit and SUPERSCRIPT^™^ III RT were purchased from Thermo Fisher Scientific, USA. Other reagents were acquired analytically from local suppliers (Sorocaba, Brazil). Cytotoxicity and genotoxicity evaluations were performed using three cell lines (Table 1) obtained from BCRJ (Cell bank of Rio de Janeiro).

**Table 1:** Cell lines used in cytotoxicity and genotoxicity assays: species, origin/code and technical characteristics

Cell line	Species	Origin	Technical characteristics
HaCat	*Homo sapiens*	BCRJ Code 0341	Tissue: Skin Cell type: keratinocytes
V79-4	*Cricetulus griséus* (Chinese Hamster)	BCRJ Code 0244	Tissue: Lung Cell type: Fibroblasts
3T3—Swiss albino	*Mus musculus* (Swiss albino)	BCRJ Code 0017	Tissue: Embrionary Cell type: Fibroblasts

### Methods

#### Obtaining of *Sclerotinia sclerotiorum* cell wall

The *Sclerotinia sclerotiorum* sclerotia were grown on potato dextrose agar (PDA) plates at room temperature and 12 h photoperiod for 7 days. After this period, mycelium discs were transferred to potato dextrose broth (PDB) and kept under stirring at 180 rpm and room temperature for 7 days. Then, the biomass was collected by filtration, lyophilized and macerated in liquid nitrogen, giving rise to a powder. Distilled water was added to the fungal cell wall powder at 5 mg mL^−1^. This mixture was centrifuged at 12,000 ×*g* for 15 min, and the presence of protein in the supernatant was evaluated by the Bradford assay [42]. The washing and centrifugation process was repeated until no proteins were detected in the supernatant. The cell wall was again lyophilized and macerated to supplement the culture broth and induce the production of enzymes from the *T. harzianum* fungus in synthesizing titanium nanoparticles with stimulation.

#### *Trichoderma harzianum* culture and biogenic synthesis of titanium nanoparticles

The culture of *T. harzianum* was performed using the commercial product Ecotrich WP (Balagro—10^10^ UFC g^−1^, according to the manufacturer). Potato dextrose agar plates were inoculated with 1 mL of *T. harzianum* suspension (200 mg mL^−1^) and kept in the dark at room temperature for 6 days. Then, mycelium discs were transferred to two different broths, potato dextrose broth supplemented with the cell wall of *S. sclerotiorum* (0.5%) as a stimulator for enzymatic activity, and potato dextrose broth without supplementation [21]. The cultures were kept in the dark at constant stirring (150 rpm) and room temperature for 12 days. Then, the biomasses were collected by filtration, transferred to ultrapure water and left under the conditions mentioned above for 72 h, followed by filtration and use of the filtrates for synthesizing the nanoparticles. Titanium IV Rutile oxide was added to the filtrates to a final concentration of 1 mM. The suspensions were kept under stirring at room temperature for 24 h to synthesize the nanoparticles [43]. Two different nanoparticles were obtained: NPTiOIVR-NS (without *S. sclerotiorum* stimulation) and NPTiOIVR-WS (with *S. sclerotiorum* stimulation).

#### Initial physico-chemical characterization of the nanoparticles

The concentration of the nanoparticle suspensions (NPs mL^−1^) was obtained by nanoparticles tracking analysis (NTA—NanoSight LM14) [44, 45]. The samples were 100-fold diluted and analysed by NanoSight LM14 and NanoSight v.2.3 software [46]. Dynamic Light Scattering (DLS) technique was performed to obtain the average hydrodynamic diameter (HD) and polydispersity index (PDI) and microelectrophoresis technique to determine zeta potential (ZP) (ZetaSizer Nano ZS 90—Malvern). The nanoparticles were two-fold diluted, and three readings were performed per sample at a fixed angle of 90º and temperature of 25 ºC. For TEM analysis, a drop of the samples was added to a copper grid and treated with a drop of 2% (v/v) aqueous uranyl solution. Then, the grid was kept at room temperature until the samples were dried entirely and later analyzed in a Zeiss transmission electron microscope -LEO 906 equipped with Olympus' CCD camera and iTEM image capture software, which operates with voltage 60 kV, with a tungsten filament. The analyses were performed at the Electronic Microscopy Laboratory of the Biology Institute of the State University of Campinas (UNICAMP), Brazil.

#### Growth of residual *T. harzianum* from the synthesis

After the synthesis, residual *T. harzianum* conidia were observed in the suspension of nanoparticles. An assay was performed to evaluate the viability of the fungus carried by the suspension. For this purpose, initially, the biogenic titanium nanoparticles were separately added to PDA plates at the concentration of 1 × 10^8^ NPs mL^−1^. To investigate the ability of the nanoparticles in stimulating the growth of *T. harzianum*, plates containing equal concentrations of nanoparticles were inoculated with commercial *T. harzianum* (0.127 mg mL^−1^). Control plates containing only commercial *T. harzianum* were also prepared. The cultures were kept at room temperature, for 7 days, with 12 h of photoperiod, and then *T. harzianum* growth was evaluated [22].

Once Trichoderma growth was observed on the plates, the kinetics of fungal growth was evaluated. Sterile filter discs embedded in the suspensions of nanoparticles (10^8^ NPs mL^−1^) were added to the center of PDA plates (triplicate), and the cultures were kept at room temperature and 12 h photoperiod. *T. harzianum* growth was monitored through photos for later kinetics evaluation using ImageJ Software. To calculate the kinetics growth, zero time (T0) was defined as the time when mycelial growth was observed exceeding the filter limits in any of the plates. The cultures were photographed at times T0 and 3, 6, 9, 12, 24, 36, 48 and 60 h after plating. The growth curve was plotted through the relative growth area of the fungus as a function of time, evaluated and calculated using Eq. 1.

1 $$Relative \,area=\frac{A(T)-A(T0)}{\mathrm{A}\left(\mathrm{F}\right)-A(T0)}$$

where, A(T) = area in the evaluated time, A(T0) = area in zero time (considered the initial mycelial growth) and A(F) = total plate area.

#### Evaluation of the activity of the nanoparticles against *Sclerotinia sclerotiorum*

To verify the activity of the biogenic titanium nanoparticles against *S. sclerotiorum *in vitro*,* PDA plates were supplemented with filtered (0.2 µm) and non-filtered suspensions of nanoparticles (10^8^ NPs mL^−1^), and *T. harzianum* (0.127 mg mL^−1^) as a control. After agar solidification, one sclerotia (resistance structure of *S. sclerotiorum*) was inoculated in the center of each plate, keeping control plates free of nanoparticles and *T. harzianum*. The cultures were kept at room temperature for 7 days, with 12 h photoperiod. After the culture period, mycelial growth and the generation of new sclerotia were observed. All tests were performed in triplicate.

#### Characterization of the selected NPTiOIV-NS

Based on the previous results obtained for both the nanoparticles, NPTiOIV-NS was selected to continue the investigations. Scanning electron microscopy (SEM) analysis of the nanoparticles' morphology was performed with a scanning electron microscope (FEI-Inspect-F50-LNNano, Campinas) at different magnifications, using a secondary electron detector (Everhart Thornley SED) operating with an acceleration voltage of 2 kV. The nanoparticle suspension was dripped onto 5 × 5 mm (4˝ Ø) previously treated (glow discharge) silicon chips. After drying, the silicon substrate was added to the sample holder with carbon glue, and thick carbon film (10 nm) deposition under an argon atmosphere was performed. Energy Dispersive Spectroscopy (EDS) was also performed to identify the elemental composition of NPTiOIV-NS [47].

The X-ray diffraction (XRD) analyses was performed using the solid nanoparticle sample obtained by freeze-drying of the aqueous suspension. The diffractogram was obtained using a Shimadzu XRD-6100 diffractometer (Laboratory of Materials FINEP 3, Federal University of São Carlos, Sorocaba) operated at 40 kV and 30 mA, with the copper emission line (λCu Kα = 1.5418 Å) as the radiation source. Scanning was performed in the angle range from 10 to 90°, at a rate of 2°/min, with a step size of 0.02°. Fourier transform infrared spectroscopy (FTIR) analyses was performed to investigate the main functional groups in the composition of the nanoparticles and *T. harzianum* filtrate. KBr tablets were prepared using a proportion of 1.5% of the solid nanoparticles. The spectra were acquired in the range from 4000 to 400 cm^−1^, at 8 cm^−1^ resolution, with 64 scans, using a JASCO FT/IR-410 spectrometer.

#### Chitinolytic assay to determine the chitinase activity of the nanoparticles

The chitinolytic activity assay was performed to detect the presence of active enzymes in the suspension. The indicative medium was prepared according to Agrawal and Kotasthane [48]. Filter paper discs soaked with the nanoparticles were placed in the center of the agar Petri dishes, which were incubated for 7 days at 25 ºC. Photos were taken 3, 5 and 7 days after incubation to record the formation of the halo of chitin degradation. The assay was performed in triplicate, using *T. harzianum* as a control.

#### Evaluation of the cytotoxicity and genotoxicity of the nanoparticles on cell lines in the presence and absence of ultraviolet radiation

The cytotoxicity of the nanoparticles was determined by the evaluation of mitochondrial activity and membrane integrity through the reduction of MTT (3-(4,5-dimethylthiazolyl-2)-2,5-diphenyltetrazolium bromide) salt and the exclusion of trypan blue dye techniques, respectively, with and without exposure to UV radiation. In the assays, the cells were plated at 0.5 × 10^5^ cells mL^−1^ on 96-well microplates and the culture was carried out until adhesion.

For the MTT assay, the cells were exposed to the filtered biogenic titanium nanoparticles (0.2 µm) at concentrations between 0.06 and 2.1 × 10^9^ NPs.mL^−1^ for 24 h [9]. At the end of the exposure period, the wells were washed with phosphate-buffered saline (PBS), the MTT solution (0.5 mg mL^−1^) was added to the wells, and the plates were incubated for 3 h, followed by the MTT removal and addition of DMSO. The absorbance was read at 540 nm (Readwell PLATE, ROBONIK). All the tests were performed in sextuplicate, and the results were analyzed by the calculation of relative viability, considering the average absorbance values obtained in the negative control as 100% viability. The exact parameters of the MTT assay were employed for the trypan blue dye exclusion assay. The cells were exposed to the filtered biogenic titanium nanoparticles for 1 h. The plates were kept in an incubator until the end of each analysis. After cell exposure, trypan blue dye solution (0.4%) was added to the cell culture (1:1 v/v), and the counting of viable and nonviable cells was performed using an optical microscope. The percentage of viable cells was calculated using Eq. 2 [49].

2 $$Cell \,viability (\%)= \frac{Number \,of \,viable \,cells}{Total \,cells (viable \,cells+nonviable \,cells)} \mathrm{X} 100$$

For the analysis in the presence of UV radiation, the cell cultures were exposed to UVC radiation 30 min after the start of the exposure to the nanoparticles (OSRAM PURITEC HNS UV-C lamp 30 W, dominant wavelength: 254 nm) for 10 min, with a distance of 12 cm from the surface of the culture media containing the cells [50, 51], and incubated again until the entire incubation period (24 h exposure).

The comet assay was performed according to the adapted methodology of Singh et al. to determine the genotoxicity of the nanoparticles [52]. The cells were exposed to the filtered nanoparticles at 1 × 10^9^ and 1 × 10^8^ NPs mL^−1^ for 1 h, followed by homogenization with low melting point agarose (0.8%) and spread on 1.5% agarose-coated slides using coverslips. All the assay was performed in triplicate. After slide preparation, cell lysis was performed at 4 ºC, then neutralization and transference to the electrophoresis chamber. The cells were kept in an electrophoresis buffer for 20 min for DNA unwind, and the electrophoresis was performed (20 min at 30 V, 300 mA and 10 W). After electrophoresis, the slides were neutralized and left to dry at room temperature overnight [9]. The slides were previously fixed for silver staining, kept in staining solution for 35 min, and left to dry at room temperature. The analyses were performed using an optical microscope by visual scoring, classifying the DNA damage into five different categories, where 0 (D0) indicates the absence of damage and 4 (D4) indicates the higher damage index [53].

#### Effects of the nanoparticles on microorganisms of agricultural importance and nitrogen-cycling bacteria

The minimal inhibitory concentration (MIC) assay to evaluate the possible effects of the nanoparticles on the development of microorganisms of agricultural importance was performed with *Bacillus thuringiensis* [54], *Pseudomonas aeruginosa* [55], *Bradyrhizobium japonicum* [56] and *Beauveria bassiana* [57]. The assays were performed in triplicate with two controls: microorganisms free of nanoparticle exposure and culture media without microorganisms. The microorganisms were incubated at 35ºC for 24 h in 96-well microplates containing *Müeller Hinton* broth supplemented with the nanoparticles in concentrations between 0.06 and 2.1 × 10^9^ NPs mL^−1^ [9]. After exposure, resazurin dye was added (6.75 mg mL^−1^), and the microplates were incubated for 24 h. Results were obtained by visual analysis, considering the colour change from blue to pink as an indicator of the viability of the microorganisms.

Molecular analysis of soil bacteria was performed to verify the possible effects of the nanoparticles on the soil microbiota, which acts in nitrogen cycling. Initially, the soil was sieved, separated into 10 g aliquots in conical tubes and exposed to 2.6 mL of nanoparticle suspensions at a concentration of 1 × 10^8^ NPs mL^−1^. A sample containing *T. harzianum* in a concentration proportional to that used in the field and negative control (only water) was also prepared [58]. Initially, DNA was extracted from a soil sample without any type of exposure (denoted soil zero) for use as a control reflecting the initial soil conditions. DNA extraction was performed using Power Soil^®^ DNA Isolation Kit (Qiagen). DNA extractions were performed 15 and 360 days after exposure, and the genetic material was quantified by fluorescence (Qubit 3.0 Fluorometer) and diluted to a final concentration of 100 ng mL^−1^. Gene quantification was performed by qPCR with specific primers (Nitrogenase reductase—*nifH*, Cu-containing nitrite reductase—*nirK*, Nitrite reductase—*nirS*, Nitric oxide reductase—*cnorB*, Nitrous oxide reductase—*nosZ* and Nitrate reductase—*narG*), in triplicate, employing Step One SYSTEM (Thermofisher), with the amplification conditions according to Maruyama et al. [59]. 16S rRNA was used as a reference gene for the relative quantification of bacterial DNA, and the DNA sample initially extracted from soil zero was used as a control.

#### Effects of the nanoparticles on soybean plants

Soybean was cultivated in plastic pots (14 cm in upper diameter, 9.5 cm in lower diameter and 10.5 cm in height) containing soil previously exposed to the nanoparticles in the proportion of 3.82 × 10^13^ NPs m^2^. Five seeds were planted per pot, with five repetitions. The exact number of pots was prepared as control with soil free of nanoparticles. The experiment was conducted in a greenhouse in a randomized design under natural light conditions for 25 days (from April 25th 2019 to May 20th 2019), with daily watering and supplementation with 50 mL of Hoagland and Arnon's nutrient solution (1 mM KH_2_PO_4_, 4 mM Ca(NO_3_)_2_.4H_2_O. 2 mM K_2_SO_4_, 4 mM (NH_4_)2SO_4_, 2 mM MgSO_4_.7H_2_O, 92.5 μM H_3_BO_3_, 18 μM MnCl_2_.4H_2_O, 1.5 μM ZnCl_2_, 0.56 μM Na_2_MoO_4_.2H_2_O, 0.66 μM CuCl_2_.2H_2_O, 100 μM FeSO_4_) twelve days after sowing [60]. After plant growth, morphological parameters were evaluated by measuring shoot and root length, leaf area, shoot and root fresh and dry weight. Hydrogen peroxide (H_2_O_2_) and malondialdehyde (MDA) were quantified in leaves and roots as markers of oxidative stress and lipid peroxidation, respectively. Initially, for the analysis of oxidative stress, 0.1 g of vegetal tissue (leaf and root) were ground to a powder with liquid nitrogen and diluted in 1 mL cold 0.2% trichloroacetic acid solution in methanol (w/v), followed by centrifugation at 13,700 xg, at 4 ºC for 5 min, collecting the supernatant for hydrogen peroxide and malondialdehyde quantification, according to Alexieva et al. [61] and Camejo et al. [62], respectively. To investigate the effects of the nanoparticles on soybean roots, the expression of genes involved in root lignification was analyzed. Total RNA was extracted from the plant roots following the methodology described by Bitencourt et al. [63]; RNA was quantified (Qubit™ RNA HS Assay Kit) and converted to complementary DNA (cDNA) by reverse transcription (SUPERSCRIPT™ III RT). Real-time PCR (StepOne thermocycler) was performed employing the ΔΔCT (2^−ΔΔCT^) method with specific primers to evaluate the expression of Phenylalanine ammonia-lyase (*PAL*), Cinnamate 4-hydroxylase (*C4H*), Cinnamyl alcohol dehydrogenase (*CAD*), Peroxidase 2 (*POD2*), Peroxidase 4 (*POD4*) and Peroxidase 7 (*POD7*) genes [64]. The β-actin gene was used as an endogenous normalizer.

### Statistical analysis

Statistical analysis was performed using ANOVA, followed by Tukey's HSD post hoc test (equal variances) and Wilcoxon's T-test, GraphPad Prism program. Statistical significance was defined as p < 0.05.

## Results and discussion

### Synthesis and initial characterization of titanium nanoparticles

**Fig. 1:**
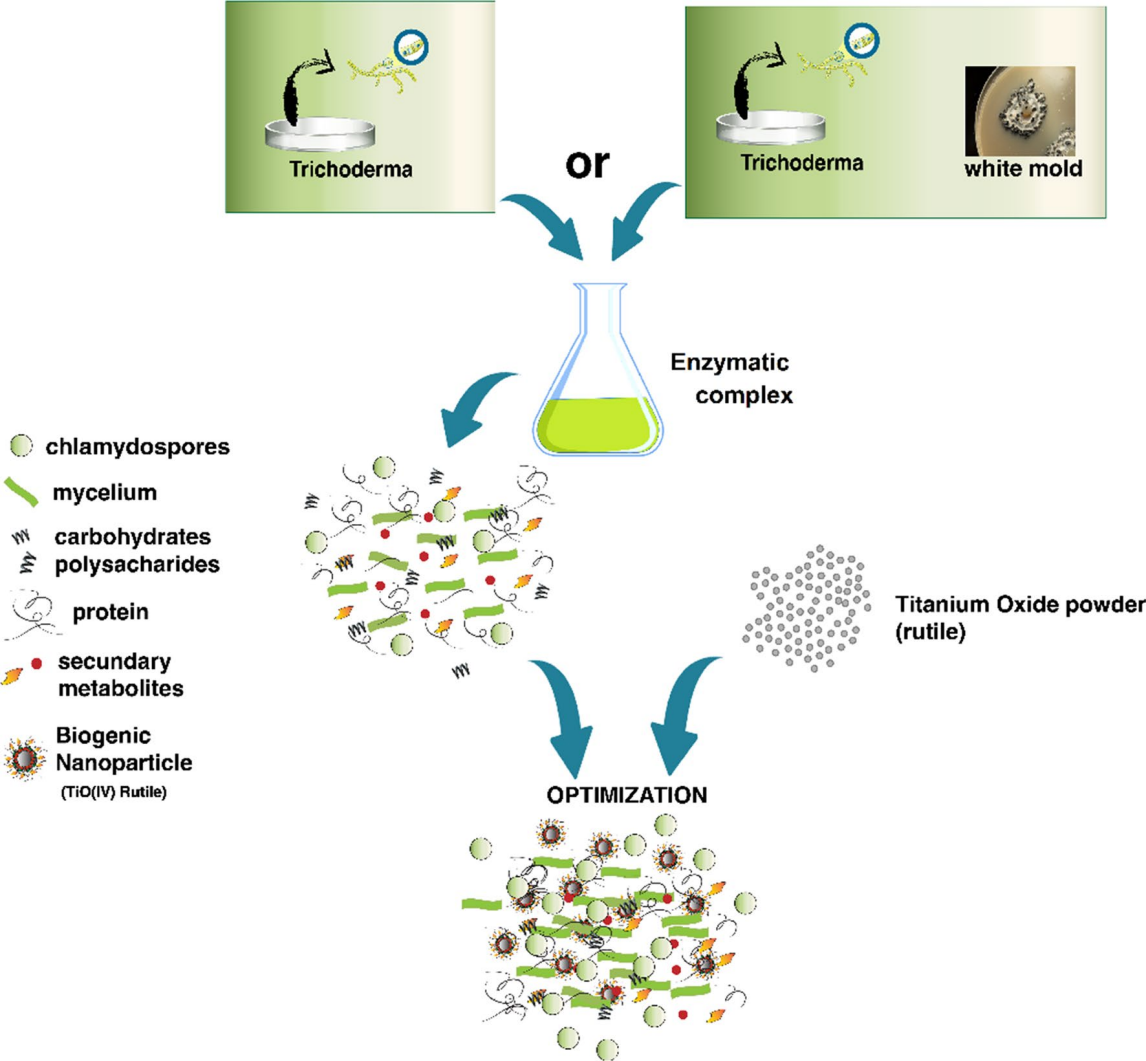
Mechanism of synthesis of titanium nanoparticles with and without supplementation with *S. sclerotiorum* cell wall suggesting the production of different biomolecules and the formation of different cappings

The biogenic synthesis of metallic nanoparticles is generally proposed as the action of metabolites from the organisms employed as reducing and stabilizing agents in the process [65, 66]. However, as the metallic precursors used in the present study are not reactive, it is possible to suppose that the synthesis occurs through the dispersion of titanium oxide in the enzymatic content of *T. harzianum* followed by the adsorption of fungal protein on the surface of the nanoparticles. In this way, suspensions containing titanium oxide nanoparticles capped with biomolecules synthesized by the fungus, free biomolecules (not adsorbed to the nanoparticles) and fungal structures such as mycelium residues, conidia and chlamydospores are obtained. Figure 1 demonstrates the possible mechanism of synthesis of titanium nanoparticles with and without supplementation with the cell wall of *S. sclerotiorum* (0.5%) and suggests the formation of different cappings.

This hypothesis suggests that biogenic nanoparticles may be synthesized from compounds that do not release ions and do not suffer enzymatic reduction, different from silver and iron oxide nanoparticles [14, 22]. In the case of the present study, the nanoparticles were synthesized with the same metallic precursor and different metabolites due to the enzymatic stimulation employing *S. sclerotiorum* cell wall (Fig. 1). According to the literature, the precursors used in the synthesis are colloids, and the proteins are probably adsorbed on the surface of previously dispersed titanium oxide nanoparticles. It is justified by the fact that the stability of biogenic nanoparticles is related to the presence of a capping [14, 67, 68].

The average HD, PDI, ZP, concentration and pH of each nanoparticle were determined right after synthesis (Table 2).

**Table 2:** Physico-chemical characterization of biogenic titanium nanoparticles

Nanoparticle	pH	HD (nm)	PDI	ZP (mV)	Concentration (NPs mL^−1^)
NPTiOIVR-NS	7.4	567 ± 58	0.741 ± 0.08	− 20 ± 3	6.8 × 10^9^ ± 1.4 × 10^9^
NPTiOIVR-WS	6.5	650 ± 46	0.535 ± 0.09	− 17 ± 1	1.9 × 10^10^ ± 3.3 × 10^9^

Regarding pH, the results showed nanoparticles with pH 7.4 and 6.5 (Table 2), considered appropriate as literature shows that pH values around 7 are adequate for agricultural applications by chemigation [69–71]. The different parameters of characterization observed when comparing the nanoparticles are probably due to the different cappings composed of protein, carbohydrates and secondary metabolites released by *T. harzianum* in the presence and absence of *S. sclerotiorum* cell wall as an enzymatic stimulator. This capping confers stability and specificity to the nanoparticles [65], showing that different conditions of synthesis may result in different interactions between the capping and the metallic core (Fig. 1).

It is possible to observe that the nanoparticles synthesized with stimulation showed a larger hydrodynamic diameter (Table 2) that may be attributed to the presence of a more significant amount or diversity of biomolecules produced by *T. harzianum* in the presence of *S. sclerotiorum* cell wall. Choudhary et al. concluded that hydrolytic enzymes and other metabolites from the medium may trigger the formation of nanoparticles with larger cappings [72]. The nanoparticles synthesized without stimulation showed higher zeta potential than those with stimulation, showing the difference in the functional groups exposed to the external surface and, consequently, the difference in the capping of each nanoparticle.

**Fig. 2:**
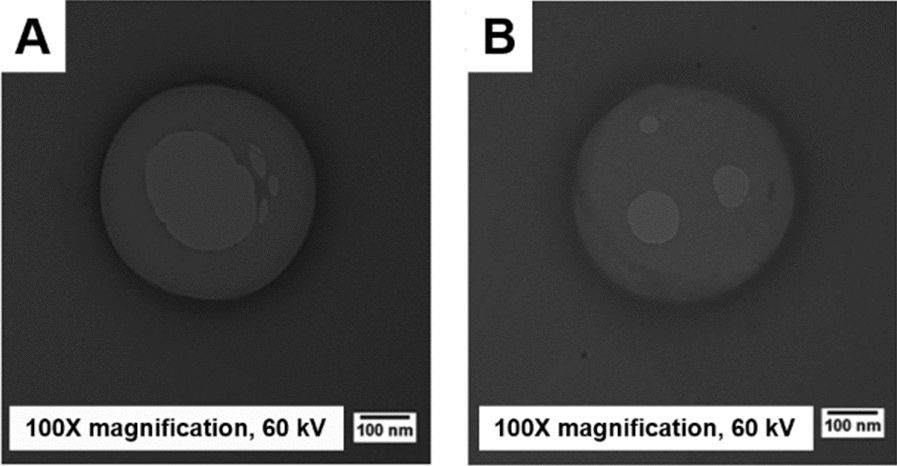
TEM micrographs of biogenic titanium nanoparticles. **A** NPTiOIVR-NS, 100 ×magnification; **B** NPTiOIVR-WS, 100 ×magnification

The analyses by TEM showed that the titanium biogenic nanoparticles have spherical morphology and smooth surface, with an average diameter of 431 ± 87 nm for NPTiOIVR-NS (Fig. 2A) and 378 ± 65 nm for NPTiOIVR-WS (Fig. 2B).

### Investigation of the presence of remaining *T. harzianum*

**Fig. 3:**
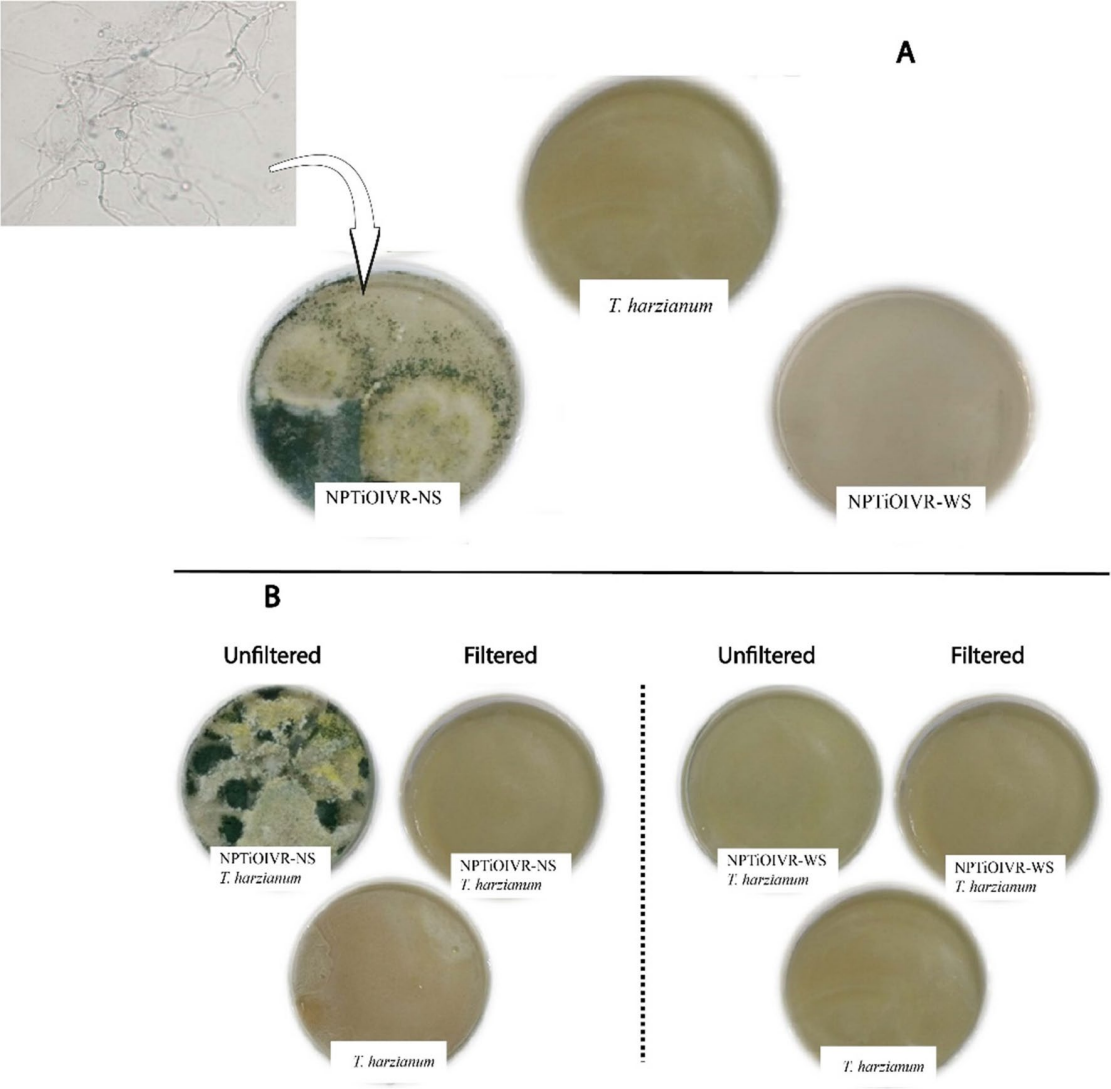
Evaluation of the growth of *T. harzianum* remaining in the suspension of biogenic titanium nanoparticles. **A** Nanoparticles without filtration of the remaining fungus; **B** Growth of remaining *T. harzianum* with and without filtration of the suspension in plates containing *T. harzianum*

Mycelial growth was observed on PDA plates inoculated with the suspension of NPTiOIVR-NS, confirming the presence of viable *T. harzianum* remaining in the suspension of nanoparticles (Fig. 3A). This growth occurs probably, due to the presence of chlamydospores or other reproductive structures in the final suspension [22]. The nanoparticles synthesized with stimulation by *S. sclerotiorum* cell wall did not show *T. harzianum* residual growth (Fig. 3A).

To verify if the biogenic titanium nanoparticles had the potential to stimulate the growth of commercial *T. harzianum* in culture, plates containing the nanoparticles and the conidia of commercial *T. harzianum* were prepared (Fig. 3B). Suspensions of filtered and unfiltered nanoparticles were used, and the results showed fast mycelial growth in the plates containing unfiltered NPTiOIV-NS (with the remaining reproductive structures). The plates containing filtered NPTiOIV-NS showed mycelial growth similar to the control plates containing only *T. harzianum*. The plates containing NPTiOIV-WS did not show fast mycelial growth, indicating that the stimulation by *S. sclerotiorum* cell wall did not enable the presence of viable reproductive propagules after synthesis. Another observation is that the filtrate employed in the synthesis did not show *T. harzianum* growth, indicating that the nanoparticles possess a potentializing effect.

A possible change in the metabolism of *T. harzianum* triggered by *S. sclerotiorum* cell wall (synthesis with stimulation) may explain the absence of viable reproductive structures in NPTiOIV-WS. Probably, the defense mechanisms of *T. harzianum* were activated as a priority, and the production of reproductive structures was converted into a secondary mechanism [73, 74], directing the metabolism to fight the pathogen instead of employing energy on development and multiplication [75]. It is possible to observe that compared to the commercial *T. harzianum* control, the plates containing commercial Trichoderma + NPTiOIVR-NS nanoparticles show more significant mycelial growth (Fig. 3B). This growth is not observed in the plate containing commercial Trichoderma + filtered nanoparticles, confirming the previous existence of reproductive structures remaining from the synthesis. In the plates containing NPTiOIV-WS with commercial *T. harzianum,* the growth was relatively the same as in the control plates. These results corroborate the hypothesis that, probably, the addition of the cell wall of the phytopathogen during *T. harzianum* growth stimulated a higher production of hydrolytic enzymes, which play a crucial role in biological control [40, 41], triggering a shift of energy resources towards the production of metabolites against the phytopathogen.

**Fig. 4:**
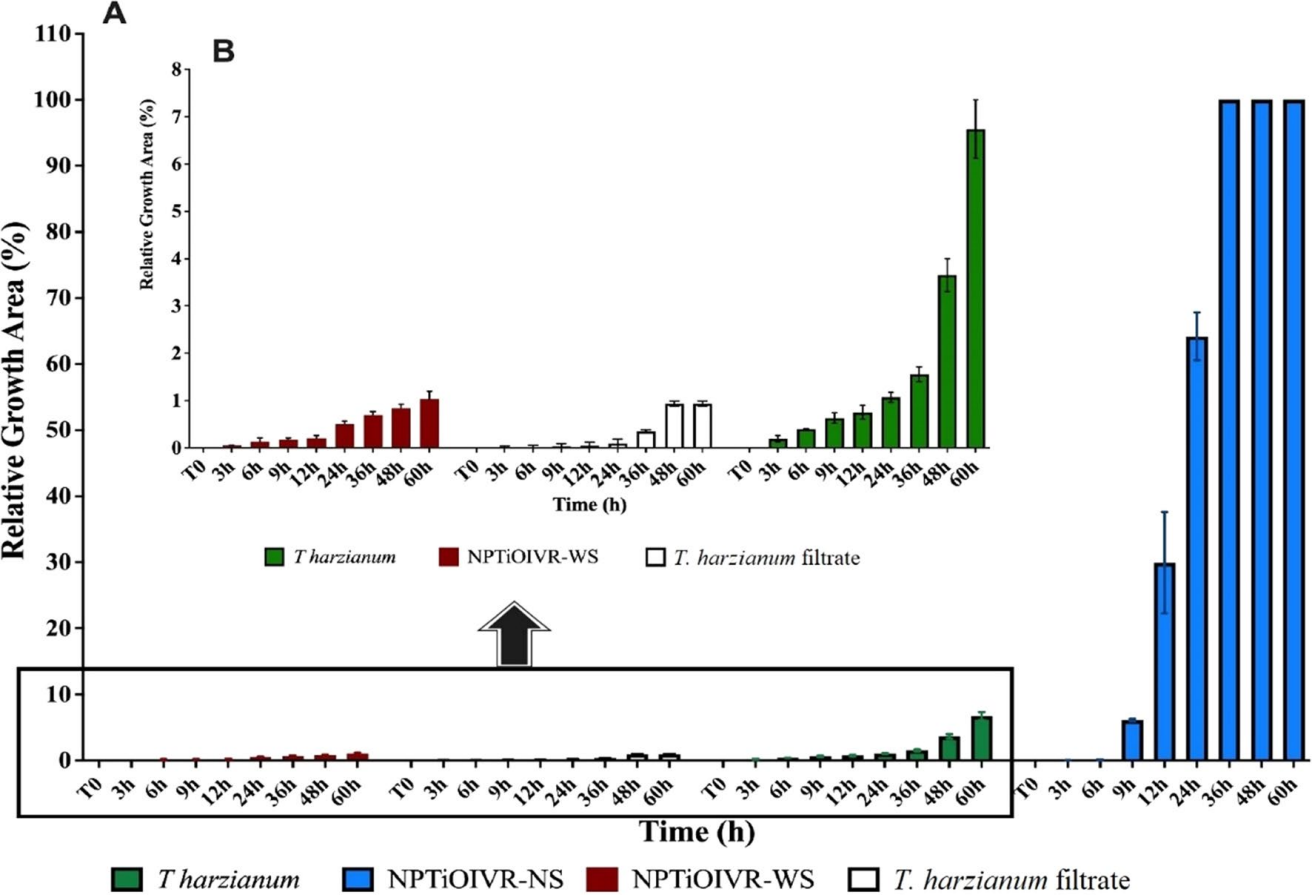
Kinetics of *T. harzianum* growth. **A** Growth area of NPTiOIVR-WS and NPTiOIVR-NS relative to commercial *T. harzianum* suspension at 0.127 mg mL^−1^ and the filtrate employed in the synthesis for comparison purposes. **B** Comparison only with NPTiOIVR-WS and commercial *T. harzianum*, highlighting the low growth in NPTiOIVR-WS

The growth kinetics was evaluated for both the nanoparticles. The results showed that NPTiOIVR-NS exhibited a significantly higher *T. harzianum* growth area with time compared to the growth of commercial *T. harzianum*. The plates containing NPTiOIVR-WS did not show *T. harzianum* mycelial growth (Fig. 4).

The reproductive structures of *T. harzianum* remaining in the suspension is not the unique explanation for the more intense development of *T. harzianum* after the synthesis of the nanoparticles since the filtrate employed in the synthesis did not show similar mycelial growth (Fig. 4). Chlamydospores are fungal structures with high resistance to stress conditions and possess an important role in fungal growth and proliferation [76, 77]. The contact of the fungus with the metallic precursor during the synthesis may be a stressor factor that triggered the development of chlamydospores as resistant structures to ensure survival. An exciting factor corroborating this hypothesis is that these structures are preferentially formed in liquid medium such as the synthesis suspension. In this way, the tests comparing commercial Trichoderma and nanoparticles, in fact, probably, compare the growth and activity of conidia and chlamydospores, respectively. Another important point to highlight is the fact that Trichoderma species produce chlamydospores, toxic substances and hydrolytic enzymes which degrade the cell wall of pathogenic fungi, favouring their survival [40, 77]. These characteristics justify the synthesis process described in this study as a scientific-based proposal, suggesting that the biological activity of the biogenic nanoparticles is triggered by a synergy between the metallic core and the capping.

### Evaluation of the influence of *Sclerotinia sclerotiorum* cell wall stimulation on the biological activity of the nanoparticles

**Fig. 5:**
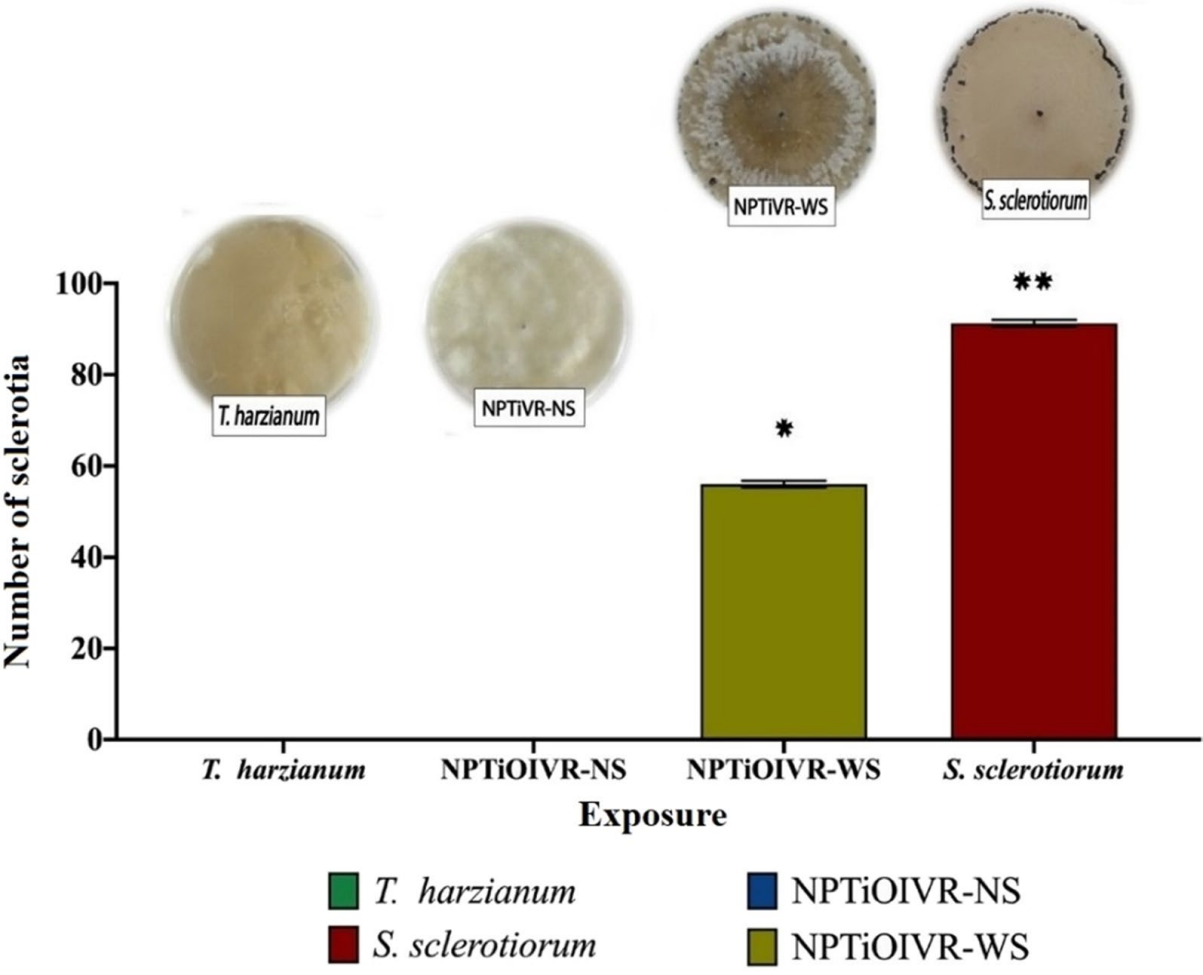
Evaluation of the activity of titanium nanoparticles for the control of *S. sclerotiorum*. Average number of new sclerotia and aspect of the culture plates showing the presence or absence of new sclerotia according to the exposure to NPTiOIV-NS, NPTiOIV-WS, *T. harzianum* and control (only culture medium)

The results of the evaluation of the biological activity of the nanoparticles in vitro showed that NPTiOIV-NS inhibited mycelial growth and the development of new sclerotia of *S. sclerotiorum* (Fig. 5).

It was possible to observe the fast growth of *T. harzianum* on the plates treated with NPTiOIV-NS probably due to the presence of residual propagules, and the colonization of the initial sclerotia, inhibiting *S. sclerotiorum* mycelial growth. In addition, NPTiOIV-NS indirectly affects *S. sclerotiorum* since it enhances the growth of *T. harzianum*, which inhibits the development of *S. sclerotiorum*. On the contrary, the cultures containing NPTiOIV-WS did not show *T. harzianum* growth and inhibition of *S. sclerotiorum*. An average amount of new sclerotia was observed (56 sclerotia), a lower number than that observed in the control plates (91.3 new sclerotia).

### Characterization of the selected NPTiOIV-NS

After the results of the biological activity of the nanoparticles, which showed that NPTiOIV-WS did not possess inhibitory potential against *S. sclerotiorum*, NPTiOIV-NS was chosen to continue the investigations due to the better performance and ease of synthesis. In view of this, a more in-depth characterization was carried out.

**Fig. 6:**
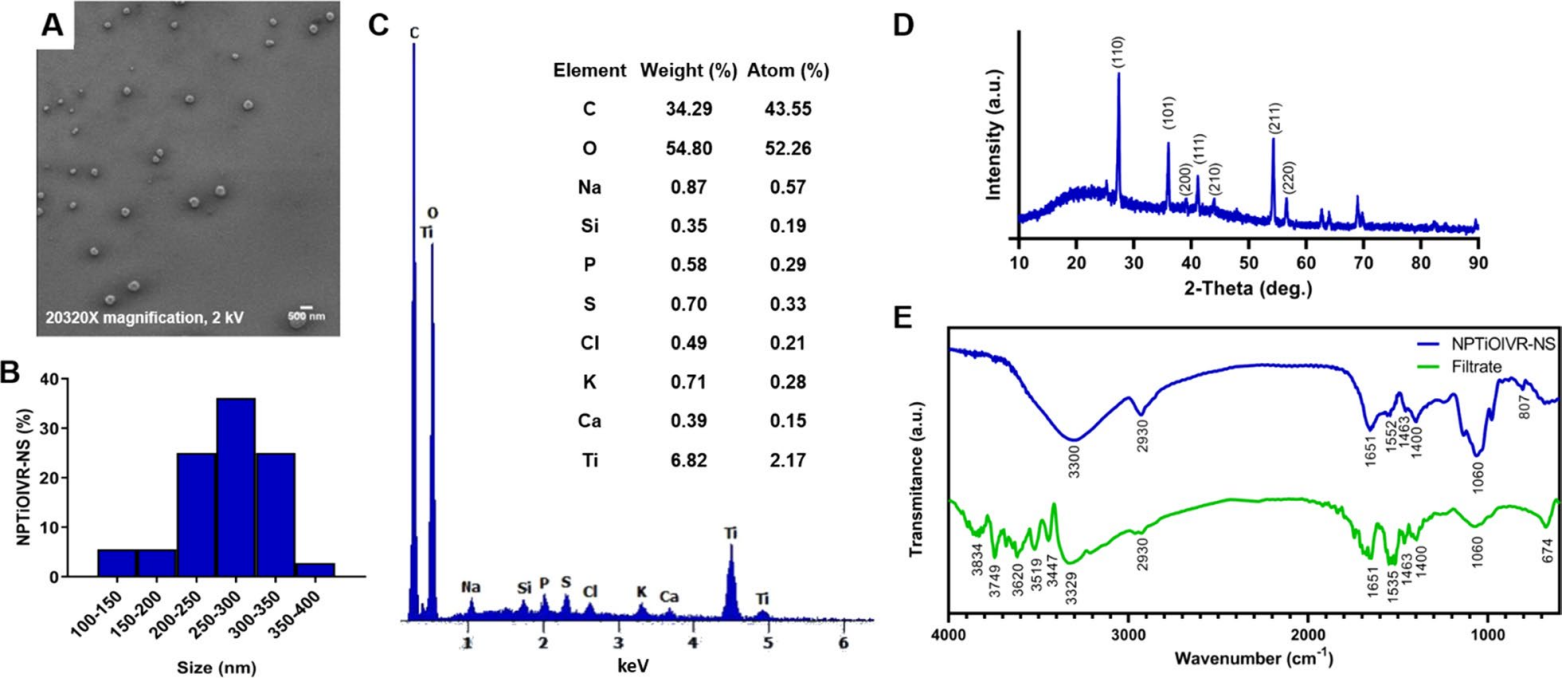
**A** SEM micrographs of NPTiOIVR-NS, 20320 ×magnification; **B** Size distribution obtained from the analysis of SEM images; **C** EDS analysis; **D** Diffractogram of XRD analysis; **E** FTIR analysis of NPTiOIVR-NS and *T. harzianum* filtrate

SEM analysis showed a size distribution between 250 and 300 nm and spherical shape (Fig. 6A and 6B). EDS analysis showed a peak characteristic of titanium, confirming the presence of the metal in elemental form in the suspension of nanoparticles. Peaks characteristic of organic compounds were detected suggesting the presence of biomolecules from *T. harzianum* associated with the nanoparticles (Fig. 6C).

XRD analysis showed diffraction peaks at 27.2º, 36.0º, 39.0º, 41.0º, 44.0º, 54.3º and 56.6º which correspond to (110), (101), (200), (111), (210), (211) and (220) crystal planes, respectively, attributed to rutile phase of titanium dioxide (rutile TiO2, JCPDS Code 751,537 [78, 79]. According to Debye–Scherrer equation, the crystal size of the nanoparticles is 21.73 nm.

The FTIR analysis showed some similar bands for *T. harzianum* filtrate and NPTiOIVR-NS. The absorption bands around 3300 cm^−1^ indicate hydroxyl (OH) groups and the N − H bond of amine groups while the band 2930 cm^−1^ may be attributed to the stretching of aliphatic C − H, 1651 cm^−1^ corresponded to stretching of the amide C = O bond and 1400 cm^−1^ may indicate a C − C vibration [14, 22, 80–82]. The band 1535 cm^−1^ attributed to amide II in the filtrate of *T. harzianum* was shifted to 1552 cm^−1^ and suffered a decrease in intensity in NPTiOIVR-NS, indicating an out-of-plane symmetric angular deformation of NH_2_. The band 1060 cm^−2^ which suffered an increase in intensity in NPTiOIVR-NS may indicate deformations of C–O [14]. These results indicate that some functional groups of the filtrate remained in the suspension of nanoparticles, however some changes in the position and intensity of the bands suggest a possible association between these groups and the metallic core.

### Chitinolytic assay to determine the chitinase activity of the nanoparticles

**Fig. 7:**
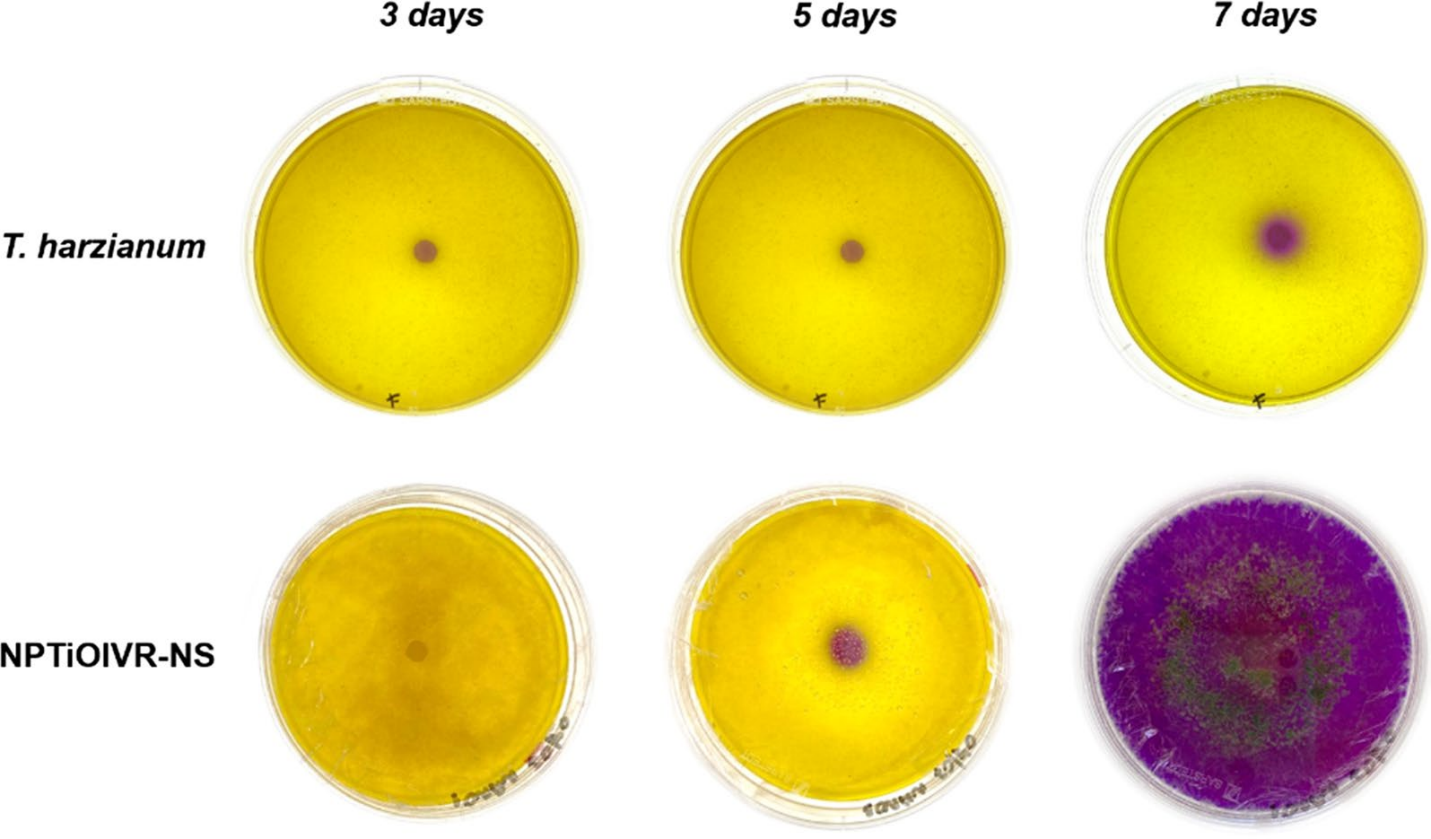
Chitinolytic activity of NPTiOIV-NS and *T. harzianum* filtrate 3, 5 and 7 days after plating

The chitinolytic activity assay showed a high activity of chitinase of NPTiOIV-NS 7 days after incubation. The medium colour change from yellow to purple was observed in the hole plate, and the mycelium growth of *T. harzianum* was also observed. At the same time, *T. harzianum* alone did not show high chitinolytic activity (Fig. 7).

Similar results were obtained by Bilesky-José et al., where a higher activity of chitinase was observed for the suspension of biogenic iron oxide nanoparticles synthesized with *T. harzianum* filtrate in comparison with *T. harzianum* alone [22]. Maruyama et al. compared the chitinolytic activity of calcium alginate microparticles containing *T. harzianum* and the non-encapsulated fungus and observed a higher activity of the microparticles, with a more intense colour change of the medium and mycelium growth [4].

### Evaluation of the cytotoxicity and genotoxicity of the nanoparticles on cell lines in the presence and absence of ultraviolet radiation

**Fig. 8:**
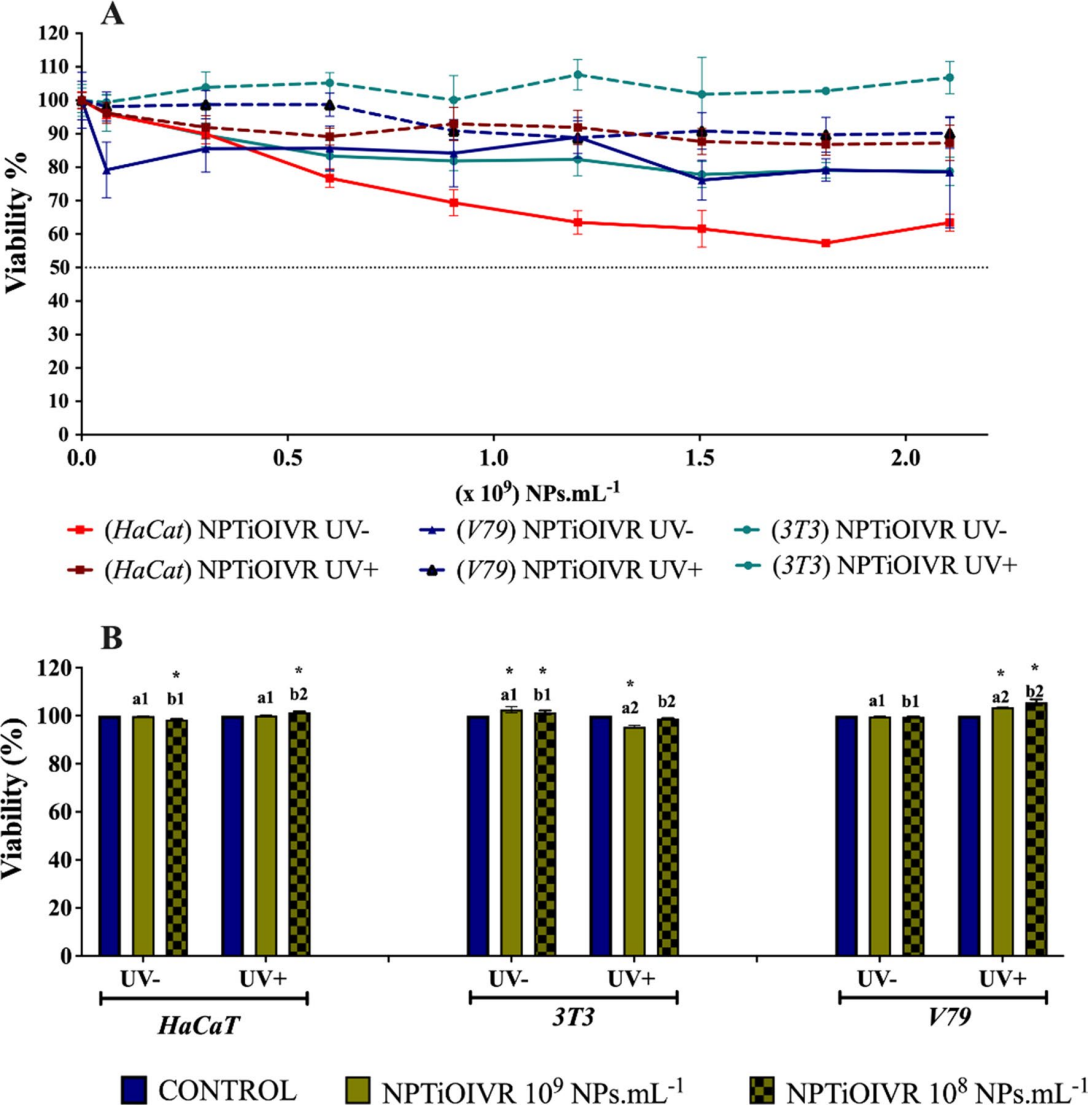
Evaluation of the cytotoxicity of biogenic titanium nanoparticles. **A** MTT assay of NPTiOIV-NS in concentrations between 0.06 and 2.1 × 10^9^ NPs mL^−1^ with different cell lines with (UV +) and without (UV− ) exposure to ultraviolet radiation. **B** Direct viability analysis of cells exposed to NPTiOIV-NS at the concentrations 1 × 10^9^ and 1 × 10^8^ NPs mL^−1^ with (UV +) and without (UV− ) exposure to ultraviolet radiation by the exclusion of trypan blue dye. *Indicates difference when compared to the control; a and b indicate comparisons between the assays without and with exposure to UVC radiation, respectively (p < 0.05)

Cytotoxicity and genotoxicity evaluations were performed since exposure to workers may occur in the case of agricultural applications. The results showed low cytotoxicity (Fig. 8) and an evident protective effect of the nanoparticles when the cells were exposed to UVC radiation. The cells showed high viability in both MTT and Trypan blue assays, without the determination of IC_50_ by MTT assay (Fig. 8A), for the cells exposed and not exposed to UVC radiation. These results indicate the low toxicity of the nanoparticles to the exposed cell lines. Park et al. demonstrated a concentration-dependent increase in the cytotoxicity of human bronchial epithelial cells exposed to titanium dioxide nanoparticles. However, IC_50_ was not obtained even at the highest concentrations, reaching just over 60% of viability in a one-hour exposure [83].

Titanium dioxide on the nanoscale is widely used in sunscreens and cosmetics due to the high index of refraction. The rutile crystalline phase is the most used for this application [84–87]. However, it is crucial to consider the possible toxic effects of this nanomaterial since some studies show impacts on health and the environment [88]. According to Morlando et al., the catalytic potential of titanium dioxide, especially at the nanoscale, may increase the production of reactive oxygen species (ROS) in the presence of UV radiation, inducing higher cytotoxicity [89]. Consequently, TiO_2_ nanoparticles are often capped with different compounds (silica, magnesium or aluminium) to prevent and reduce reactions to UV exposure [86, 90, 91]. However, these cappings are not widely studied, and few studies compare the toxicity of different TiO_2_ nanomaterials. According to Hamzeh and Sunahara, significant differences in cytotoxicity and genotoxicity were found between nano-TiO_2_ and micro-TiO_2_, as well as between capped and uncapped nano-TiO_2_ [92].

In the present study, an increase in relative cell viability of the three cell lines was observed with the exposure to the nanoparticles in the presence and absence of UVC radiation, mainly HaCat and 3T3 cell lines (Fig. 8A). It suggests that the nanoparticles may have a protective effect against the damage to the cellular metabolism of the MTT salt caused by radiation. The evaluation of direct cell viability by excluding trypan blue showed that the nanoparticles did not cause cytotoxic effects to the exposed cell lines at the concentrations of 1 × 10^8^ e 1 × 10^9^ NPs mL^−1^, with and without exposure to UVC radiation (Fig. 8B). Similar results were found by Hanot-Roy et al., that exposed lung carcinoma cells to titanium dioxide nanoparticles at a wide range of concentrations (5, 200 and 800 µg mL^−1^) and did not observe significant cytotoxic levels [93]

Therefore, the viability results indicate that the capping formed during the synthesis of the nanoparticles had a similar effect to that presented in some studies where the cappings are added to the nanoparticles to prevent the reaction with UV radiation. It is plausible to consider that the capping from *T. harzianum* metabolites had an essential role in reducing the photocatalytic action of the nanoparticles, corroborating with a study that reports a lower photocatalytic activity of titanium dioxide nanoparticles capped with the natural polymer chitosan [89].

**Fig. 9:**
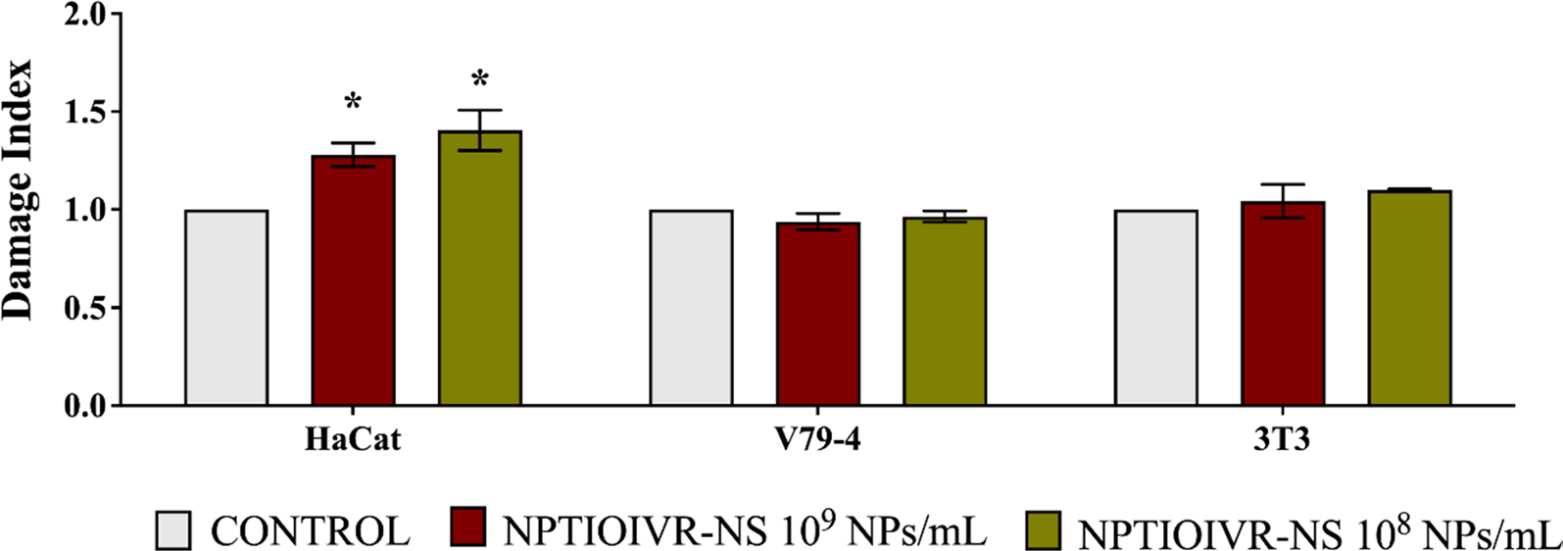
Evaluation of the genotoxicity of the biogenic titanium nanoparticles at the concentrations of 1 × 10^9^ and 1 × 10^8^ NPs mL. * Indicate significant difference (p < 0.05)

The genotoxic potential of titanium nanoparticles was evaluated by comet assay using HaCat, 3T3 and V79-4 cell lines. The results did not show an increased damage index for V79-4 and 3T3 cell lines in comparison with control, whereas HaCat cells showed higher sensitivity to both concentrations of nanoparticles (Fig. 9).

Bhattacharya et al. reported the absence of genotoxicity after lung fibroblasts and bronchial epithelium cells were exposed to anatase titanium oxide nanoparticles [94]. In contrast, the results reported by Patel et al. showed a significant increase in DNA damage of blood cells exposed to titanium dioxide nanoparticles obtained with chemical synthesis at the concentrations of 25, 75 and 125 µM [95]. A similar response was reported by Armand et al., where low concentrations of chemical titanium dioxide nanoparticles, with prolonged exposure, triggered genotoxic effects on lung carcin*o*ma cells [96]. Koca and Duman reported a dose and time-dependent DNA damage caused by biogenic titanium nanoparticles synthesized from the leaf extract of *Mentha aquatica* [97].

These results demonstrate the importance of investigating the toxicity of nanoparticles and their relation with the physicochemical characteristics. According to the route of synthesis and the features and properties of the nanoparticles, unique behaviors in regard to toxicity may be observed.

### Effects of the nanoparticles on microorganisms of agricultural importance and nitrogen-cycling bacteria

The possible effects of the nanoparticles on the microorganisms of agricultural importance *Bacillus thuringiensis*, *Pseudomonas aeruginosa*, *Bradyrhizobium japonicum* and *Beauveria bassiana* were evaluated. No inhibitory effects were observed at the concentration of 2.1 × 10^9^ NPs mL^−1^.

**Fig. 10:**
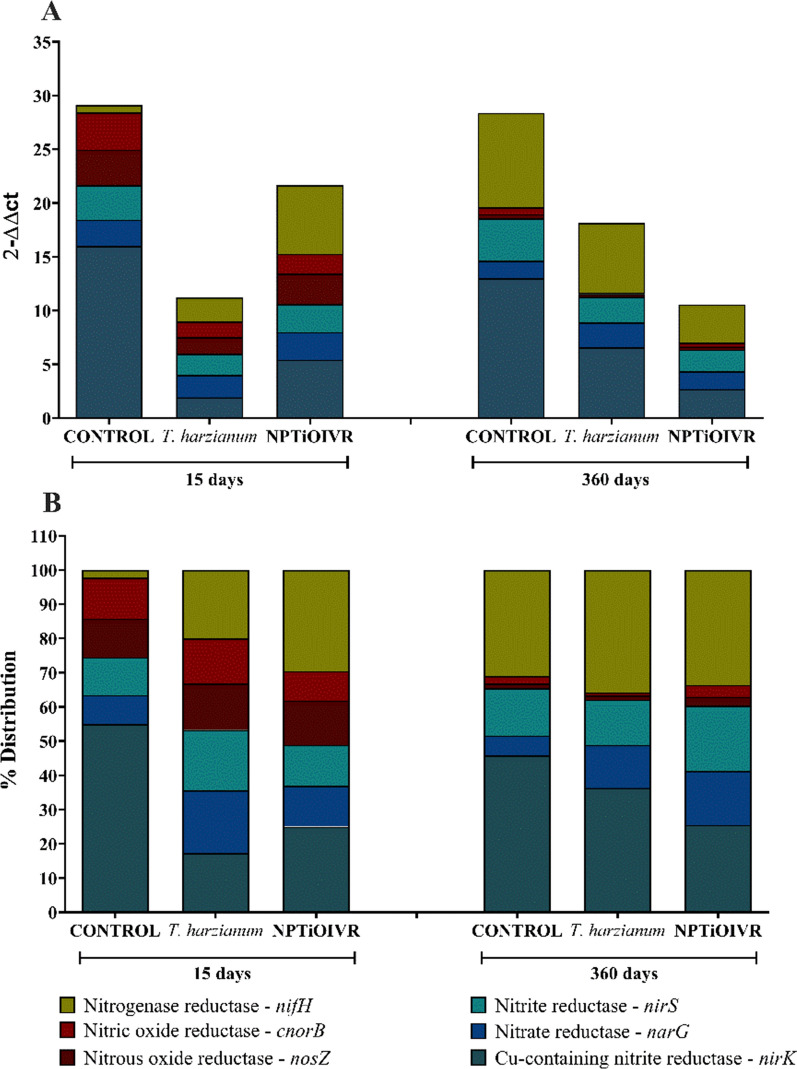
Molecular analyses of the genes of bacteria involved in the nitrogen cycle in soil exposed to NPTiOIVR-NS at the concentration 1 × 10^8^ NPs mL^−1^ and *T. harzianum* at the concentration indicated for field application. **A** Gene quantification; **B** Gene proportions

Regarding the molecular analysis of the genes of the bacteria involved in the nitrogen cycle by qPCR, 15 and 360 days after exposure, it was possible to observe a decrease in the number of bacteria in the soil exposed to NPTiOIVR-NS as well as in the soil exposed to *T. harzianum* (Fig. 10A).

The proportion of bacteria quantified in soil samples (Fig. 10B) shows that the soil exposed to NPTiOIVR-NS had similarities to the soil exposed to commercial *T. harzianum* during all the periods. This fact may be explained by the presence of remaining fungus in the suspension of nanoparticles. It is possible to observe an increase in the quantity of nitrogen-fixing bacteria (*nifH* gene) and a decrease in the number of denitrifying bacteria (*nirK* gene) compared with the control 15 days after exposure. The difference in the proportions of the genes in both the soils exposed to the nanoparticles and *T. harzianum* in comparison with control decreased 360 days after exposure, indicating a possible recovery. However, the quantification shows a decrease in the quantity of bacteria in the soil exposed to NPTiOIVR-NS, which may be due to a possible accumulation of ammonia in the soil, changing pH and causing toxicity to the bacteria [98]. Similar results were reported by Simonin et al., that observed a sequence of adverse effects on denitrifying microorganisms after a high impact of titanium dioxide nanoparticles on nitrifying bacteria, however, this effect was only observed in soils exposed to commercial *T. harzianum *[98]**.**

### Effects of the nanoparticles on soybean plants

**Fig. 11:**
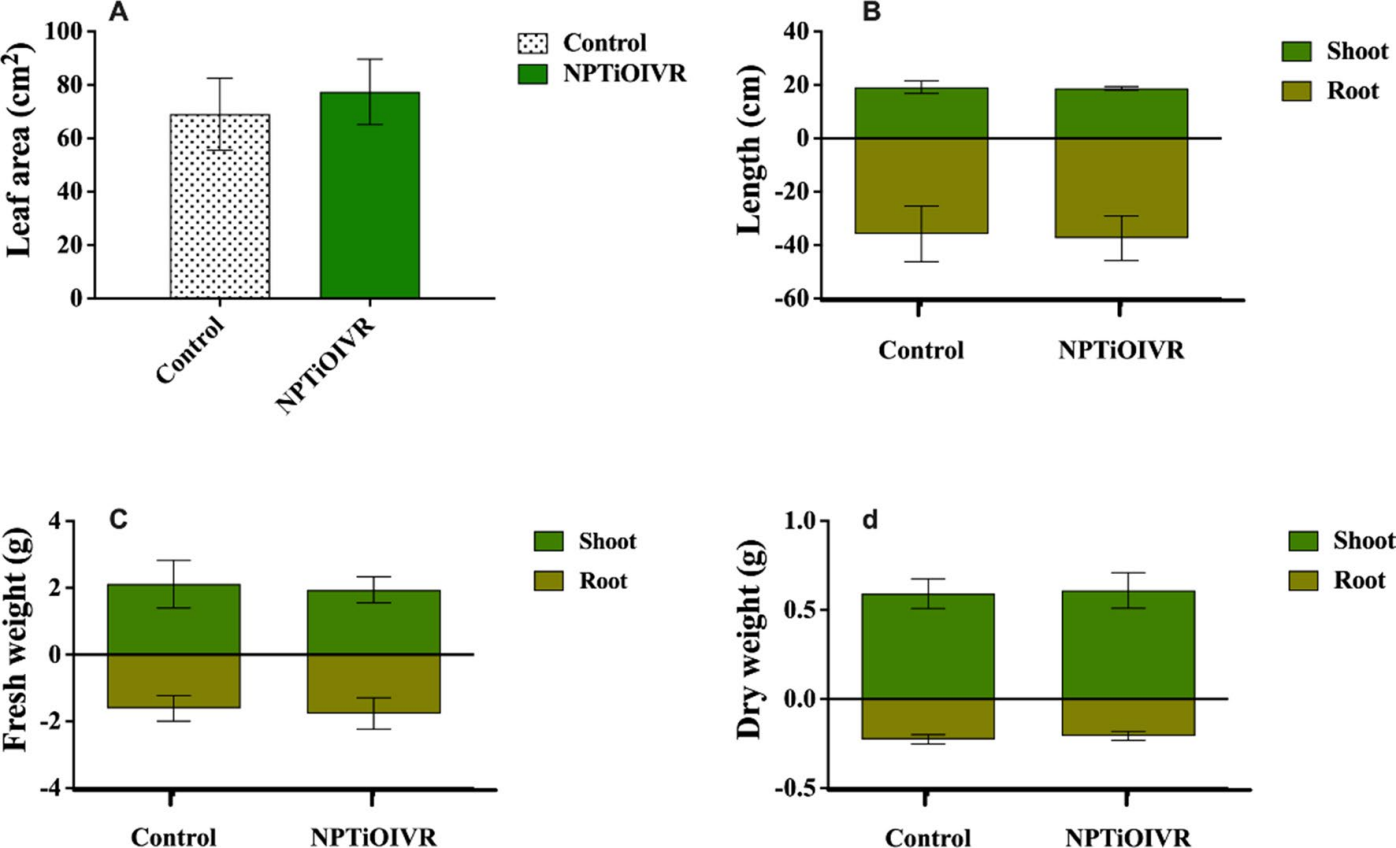
Morphological analysis of soybean plants cultivated in soil exposed to NPTiOIVR-NS at 3.82 × 10^13^ NPs m^2^. **A** Leaf area; **B** Shoot and root length; **C** Shoot and root fresh weight; **D** Shoot and root dry weight

**Fig. 12:**
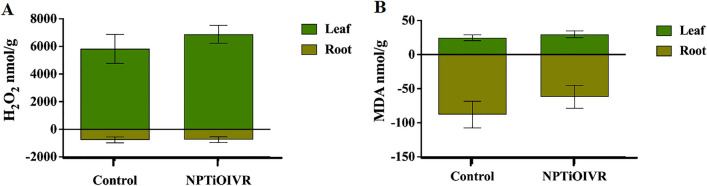
Analysis of oxidative stress and lipid peroxidation markers in the soybean plants cultivated in soil exposed to NPTiOIVR-NS in the proportion 3.82 × 10^13^ NPs m^2^. **A** Hydrogen peroxide (H_2_O_2_); **B** Malondialdehyde (MDA)

The morphological analysis of the plants exposed to NPTiOIVR-NS may be used as an indication of the phytotoxicity of nanoparticles. It is possible to observe that the nanoparticles did not cause significant changes in leaf area (Fig. 11A), shoot and root length (Fig. 11B), as well as in the fresh and dry weight of shoot and root of the soybean plants (Figs. 11C, D). Negative influences were not observed when the plants were cultivated in the soil containing the nanoparticles (Figs. 11, 12).

Several studies show that exposure to TiO_2_NPs does not cause significant changes in the shoot and root length of plants such as tomato, fennel, wheat, cucumber, corn and soybean [15, 99–102]. Regarding the biomass of the plants exposed to titanium nanoparticles, some studies report the absence of significant changes [101, 102] and other studies report increases in biomass until a limiting concentration and decreases at higher concentrations [99, 100, 103], in contrast with the results found in the present study. Mahmoodzadeh et al. reported an increase in the germination and growth of canola plantlets even at high concentrations of TiO_2_NPs (1200 and 1500 mg L^−1^) [104], which may be due to the generation of ROS triggered by the nanoparticles and as a consequence, the increase of seed resistance to stress and the promotion of greater uptake of water and oxygen, necessary for the increase of growth rate [105].

Regarding oxidative stress and lipid peroxidation, no significant changes were observed in the levels of hydrogen peroxide (H_2_O_2_) and malondialdehyde (MDA) in the leaves and roots of the plants cultivated in the soil exposed to NPTiOIVR-NS (Fig. 12).

Some studies report the increase in ROS generation caused by titanium nanoparticles triggering oxidative stress and possible toxicity. Song et al. reported a concentration-dependent increase in the oxidative stress and consequent lipid peroxidation of *Lemna minor* aquatic plants exposed to TiO_2_NPs [103]. In contrast, Foltête et al. did not observe oxidative stress when exposed *Vicia faba* plants to titanium nanoparticles capped with aluminium hydroxide and dimethicone film for sunscreen applications [106].

**Fig. 13:**
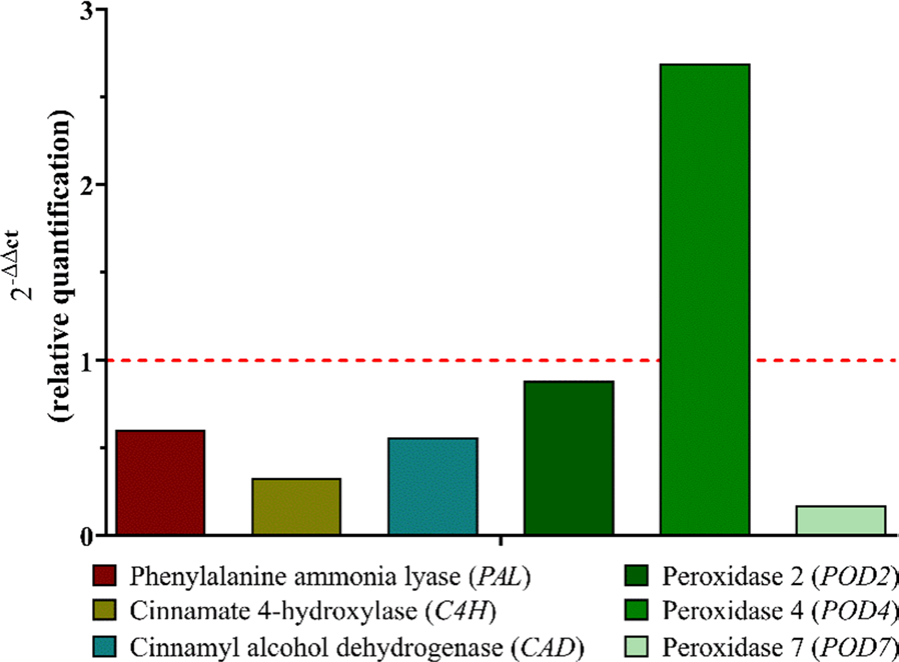
Effects of NPTiOIVR-NS in the expression of lignification-related genes that encode the enzymes phenylalanine ammonia-lyase (*PAL*), cinnamate 4-hydroxylase (*C4H*), cinnamyl alcohol dehydrogenase (*CAD*), peroxidase 2 (*POD2*), peroxidase 4 (*POD4*) and peroxidase 7 (*POD7*) of soybean roots (n = 5). The red dotted line represents the quantification of the negative control

The analysis of lignification-related genes in the roots of soybean plants showed an increase in the expression of *POD4* gene (Fig. 13).

Peroxidase 4 (*POD4*) is an enzyme in the group of class III peroxidases which have an essential role in plant physiological processes such as root lignification and other defense mechanisms against abiotic stress [107]. An increase in these enzymes' activity and gene expression probably indicates the activation of plant defense mechanisms against significant damage [108, 109]. Few studies investigate the effects of metallic nanoparticles on the expression of plant lignification-related genes. In accordance with our study, Nair and Chung observed an increase in the expression of *POD4* gene in the roots of soybean plants exposed to copper oxide nanoparticles, indicating major lignification as a defense mechanism [64]. Cunha-Lopes et al. reported an increase in root and steam lignin content and an increase in the activity of POD enzymes in soybean plants exposed to iron oxide nanoparticles compared with the control, accompanied by a decrease in the activity of PAL enzymes [108].

In the present study, the results of the plant analysis suggest that NPTiOIVR-NS do not trigger oxidative stress on soybean, corroborating with the results of plant morphology and the evaluations of toxicity on animal cell lines. The absence of adverse effects may be due to the capping of biomolecules from *T. harzianum*.

## Conclusion

The production of biogenic nanoparticles using *Trichoderma harzianum* as a reducing and stabilizing agent and TiO(IV) rutile as a metallic precursor was successful, with differences between the nanoparticles produced with and without stimulation by *S. sclerotiorum* cell wall. The presence of residual propagules, mainly hyphae and chlamydospores, was observed in the final suspension of titanium nanoparticles without stimulation and, according to the plan, it enhanced *T. harzianum* growth. It was also possible to observe that the filtrate of *T. harzianum* employed in the synthesis was insufficient to increase *T. harzianum* growth, indicating the enhancing effect of NPTiOIVR-NS.

NPTiOIVR-NS did not induce cytotoxicity on the tested cell lines, even at the highest concentrations with exposure to ultraviolet radiation. This result may be related to the presence of the capping from the fungus employed in the synthesis. Morphological changes and oxidative stress were not observed in soybean plants cultivated in the soil exposed to the nanoparticles. Regarding the effects on microorganisms of agricultural importance, no inhibition was observed on *Bacillus thuringiensis*, *Pseudomonas aeruginosa*, *Bradyrhizobium japonicum* and *Beauveria bassiana*, and minor alterations were observed in the quantification and proportion of bacterial genes involved in the soil nitrogen cycle.

The results of this study suggest that the presence of titanium nanoparticles may be responsible for the stimulation of *T. harzianum* multiplication. It is likely that the production of biogenic nanoparticles was an essential factor in stimulating or maintaining structures that are important for biological control. This may be an essential strategy to stimulate the growth of biocontrol organisms to promote more sustainable agriculture.

## Data Availability

The datasets used and analyzed during the current study are available from the corresponding author on reasonable request.

## References

[1] Liu C, Zhou H, Zhou J (2021). The applications of nanotechnology in crop production. Molecules.

[2] RUI M (2016). Iron oxide nanoparticles as a potential iron fertilizer for peanut (*Arachis*
*hypogaea*). Frontiers Plant Sci.

[3] Fatima F, Hashim A, Anees S (2021). Efficacy of nanoparticles as nanofertilizer production: a review. Environ Sci Pollut Res.

[4] Maruyama C, Bilesky-Jose N, Lima R, Fraceto LF (2020). Encapsulation of *Trichoderma*
*harzianum* preserves enzymatic activity and enhances the potential for biological control. Front Bioeng Biotechnol.

[5] Mali SC, Raj S, Trivedi R (2020). Nanotechnology a novel approach to enhance crop productivity. Biochem Biophys Rep.

[6] Wang CY, Yang J, Qin JC, Yang YW (2021). Eco-friendly nanoplatforms for crop quality control, protection, and nutrition. Adv Sci.

[7] WANG S (2016). A novel upconversion luminescence turn-on nanosensor for ratiometric detection of organophosphorus pesticides. RSC Adv.

[8] Sharma P, Pandey V, Sharma MMM, Patra A, Singh B, Mehta S, Husen A (2021). A review on biosensors and nanosensors application in agroecosystems. Nanoscale Res Lett.

[9] Pasquoto-stigliani T, Campos EVR, Oliveira JL, Silva CMG, Bilesky-José N (2017). Nanocapsules containing neem (*Azadirachta*
*Indica*) oil development characterization, and toxicity evaluation. Sci Rep.

[10] Oliveira JL (2018). Geraniol encapsulated in chitosan/gum arabic nanoparticles: a promising system for pest management in sustainable agriculture. J Agricult Food Chem.

[11] Pascoli M, An ecotoxicological perspective (2019). Neem oil based nanopesticide as an environmentally-friendly formulation for applications in sustainable agriculture. Sci Total Environ.

[12] Oliveira JL, Fraceto LF, Bravo A, Polanczyk RA (2021). Encapsulation strategies for *Bacillus*
*thuringiensis*: from now to the future. J Agric Food Chem.

[13] Dam P, Paret ML, Mondal R, Mondal AK (2023). Advancement of noble metallic nanoparticles in agriculture: a promising future. Pedosphere.

[14] Guilger-Casagrande M, Germano-Costa T, Bilesky-José N, Pasquoto-Stigliani T, Carvalho L, Fraceto LF, Lima R (2021). Influence of the capping of biogenic silver nanoparticles on their toxicity and mechanism of action towards *Sclerotinia*
*sclerotiorum*. J Nanobiotechnol.

[15] Andersen CP (2016). Germination and early plant development of ten plant species exposed to titanium dioxide and cerium oxide nanoparticles. Environ Toxicol hem.

[16] Lyu S, Wei X, Chen J, Wang C, Wang X, Pand D (2017). Titanium as a beneficial element for crop production. Front Plant Sci.

[17] Mathew SS, Sunny NE, Shanmugam V (2021). Green synthesis of anatase titanium dioxide nanoparticles using *Cuminum cyminum* seed extract; effect on Mung bean (*Vigna*
*radiata*) seed germination. Inorg Chem Commun.

[18] Sidhu AK, Verma N, Kaushal P (2022). Role of biogenic capping agents in the synthesis of metallic nanoparticles and evaluation of their therapeutic potential. Front Nanotechnol.

[19] Ballottin D (2016). Elucidating protein involvement in the stabilization of the biogenic silver nanoparticles. Nanoscale Res Lett.

[20] Guilger M (2017). Biogenic silver nanoparticles based on *Trichoderma*
*harzianum*: synthesis characterization, toxicity evaluation and biological activity. Sci Rep.

[21] Guilger-Casagrande M, Germano-Costa T, Pasquoto-Stigliani T, Fraceto LF, Lima R (2019). Biosynthesis of silver nanoparticles employing *Trichoderma*
*harzianum* with enzymatic stimulation for the control of *Sclerotinia*
*sclerotiorum*. Sci Rep.

[22] Bilesky-José N, Maruyama C, Germano-Costa T, Campos E, Carvalho L, Grillo R, Fraceto LF, Lima R (2021). Biogenic α-Fe2O3 nanoparticles enhance the biological activity of trichoderma against the plant pathogen *Sclerotinia*
*sclerotiorum*. ACS Sustain Chem Eng.

[23] Ramírez-Valdespino CA, Orrantia-Borunda E (2021). Trichoderma and nanotechnology in sustainable agriculture: a review. Frontiers Fungal Biol.

[24] Sood M, Kapoor D, Kumar V, Sheteiwy MS, Ramakrishnan M, Landi M, Araniti F, Sharma A (2020). Trichoderma: the "secrets" of a multitalented biocontrol agent. Plants.

[25] BononI L, Chiaramonte JB, Pansa CC, Moitinho MA, Melo IS (2020). Phosphorus-solubilizing *Trichoderma* spp from amazon soils improve soybean plant growth. Sci Rep.

[26] Alfiky A, Weisskopf L (2021). Deciphering *Trichoderma*–plant-pathogen interactions for better development of biocontrol applications. J Fungi.

[27] Sarangi S, Swain H, Adak T, Bhattacharyya P, Mukherjee AK, Kumar G, Mehetre ST (2021). Trichoderma-mediated rice straw compost promotes plant growth and imparts stress tolerance. Environ Sci Pollut Res.

[28] O’Sullivan CA, Belt K, Thatcher LF (2021). Tackling control of a cosmopolitan phytopathogen: sclerotinia. Front Plant Sci.

[29] Xu L, Li G, Jiang D, Chen W (2018). *Sclerotinia*
*sclerotiorum*: an evaluation of virulence theories. Annu Rev Phytopathol.

[30] Asad, S. A. 2022 Mechanisms of action and biocontrol potential of Trichoderma against fungal plant diseases—A review. Ecological Complexity. 49 100978

[31] Mironenka J, Rózalska S, Sobón A, Bernat P (2021). Trichoderma harzianum metabolites disturb *Fusarium*
*culmorum* metabolism: metabolomic and proteomic studies. Microbiol Res.

[32] Liu Q, Meng X, Li T, Raza W, Liu D, Shen Q (2020). Possible role of increasing nutrient availabilities the growth promotion of peppers (*Capsicum*
*annuum* L) by *Trichoderma*
*guizhouense* NJAU4742-based biological organic fertilizer. Microorganisms.

[33] Wang H, Zhang R, Mao Y, Jiang W, Chen X, Shen X, Yin C, Mao Z (2022). Effects of *Trichoderma*
*asperellum* 6S–2 on apple tree growth and replanted soil microbial environment. J Fungi.

[34] Morán-Diez ME, Alba AEM, Rubio MB, Hermosa R, Monte E (2021). Trichoderma and the plant heritable priming responses. J Fungi.

[35] Swain H, Adak T, Mukherjee AK, Sarangi S, Samal P, Khandual A, Jena R, Bhattacharyya P, Naik SK, Mehetre ST, Baite MS, Sunil Kumar M, Zaidi NW (2021). Biopriming With *Trichoderma* strains isolated from tree bark improves plant growth, antioxidative defense system in rice and enhance straw degradation capacity front. Microbiol.

[36] Marra R, Lombardi N, Derrico G, Troisi J, Scala G, Vinale F (2019). Application of *Trichoderma* strains and metabolites enhances soybean productivity and nutrient content. J Agric Food Chem.

[37] Mansoor A, Khurshid Z, Khan MT, Mansoor E, Butt FA, Jamal A, Palma PJ (2022). Medical and dental applications of titania nanoparticles: an overview. Nanomaterials.

[38] Satti SH, Raja NI, Javed B, Akram A, Mashwani ZR, Ahmad MS, Ikram M (2021). Titanium dioxide nanoparticles elicited agro-morphological and physicochemical modifications in wheat plants to control *Bipolaris* sorokiniana. PLoS ONE.

[39] Raliya R, Biswas P, Tarafdar JC (2015). TiO2 nanoparticle biosynthesis and its physiological effect on mungbean (*Vigna*
*radiata* L). Biotechnol Rep.

[40] Geraldine AM (2013). Cell wall-degrading enzymes and parasitism of sclerotia are key factors on field biocontrol of white mold by *Trichoderma* spp. Biol Control.

[41] Qualhato TF (2013). evaluation of antagonism and hydrolytic enzyme production mycoparasitism studies of *Trichoderma* species against three phytopathogenic fung. Biotechnol Lett.

[42] Bradford MM (1976). A rapid and sensitive method for the quantification of microgram quantities of protein utilizing the principle of protein-dye binding. Anal Biochem.

[43] Kirthi AV (2011). Biosynthesis of titanium dioxide nanoparticles using bacterium Bacillus subtilis. Mater Lett.

[44] Djurišić AB (2015). Toxicity of metal oxide nanoparticles: mechanisms, characterization, and avoiding experimental artefacts. Small J.

[45] Hole P, Hodoroaba VD, Unger WES, Shard AG (2019). Particle Tracking Analysis (PTA). Characterization of nanoparticles measurement processes for nanoparticles.

[46] Monteiro RA, Camara MC, Oliveira JL (2021). Zein based-nanoparticles loaded botanical pesticides in pest control: An enzyme stimuli-, p. responsive approach aiming sustainable agriculture. J Hazard Mater.

[47] Mittal N, Kaur G (2019). Investigations on polymeric nanoparticles for ocular delivery. Adv Polym Technol.

[48] Agrawal T, Kotasthane AS (2012). Chitinolytic assay of indigenous trichoderma isolates collected from different geographical locations of Chhattisgarh in Central India. Springerplus.

[49] Kamiloglu S, Sari G, Ozdal T, Capanoglu E (2020). Guidelines for cell viability assays. Food Frontiers.

[50] Cordeiro ACS, Leite SGF, Dezotti M (2004). Inativação por oxidação fotocatalítica de Escherichia coli e Pseudomonas sp. Quim Nova.

[51] George S (2014). Differential effect of solar light in increasing the toxicity of silver and titanium dioxide nanoparticles to a fish cell line and Zebra Fish embryos. Environ Sci Technol.

[52] Singh NP (1988). A simple technique for quantitation of low levels of DNA damage in individual cells. Experimen Cell Res.

[53] Collins AR, Fleming IM, Gedik CM (1994). In vitro repair of oxidative and ultraviolet-induced DNA damage in supercoiled nucleoid DNA by human cell extract. Biochimica Biophysica Acta Gene Struct Express.

[54] Qi J (2016). Potential of entomopathogenic Bacillus thuringiensis as plant growth promoting rhizobacteria and biological control agents for tomato *Fusarium* wilt. Int J Environ Agricult Res.

[55] Ghadamgahi F, Tarighi S, Taheri P, Saripella GV, Anzalone A, Kalyandurg PB, Catara V, Ortiz R, Vetukuri RR (2022). Plant growth-promoting activity of *Pseudomonas*
*aeruginosa* FG106 and Its ability to act as a biocontrol agent against potato. Tomato Taro Pathogens Biol.

[56] Meena RS (2018). Response and interaction of Bradyrhyzobium japonicum and arbuscular mycorrhizal fungi in the soybean rhizosphere. Plant Growth Regul.

[57] McKINNON AC (2017). Beauveria bassiana as an endophyte: a critical review on associated methodology and biocontrol potential. Biocontrol.

[58] Hjelmso MH, Hansen LH, Baelum J, Feld L, Holben WE, Jacobsen CS (2014). Highresolution melt analysis for rapid comparison of bacterial community compositions. Appl Environ Microbiol.

[59] Maruyama CR, Guilger M, Pascoli M, Bilesky-Jose N, Abhilash PC, Fraceto LF, Lima R (2016). Nanoparticles based on chitosan as carriers for the combined herbicides imazapic and imazapyr. Sci Rep.

[60] Hoagland DR, Arnon DI (1950). The water culture method for growing plants without soil. berkeley: california agricultural experiment station. Circular.

[61] Alexieva V (2001). The effect of drought and ultraviolet radiation on growth and stress markers in pea and wheat. Plant Cell Environ.

[62] Camejo G, Wallin B, Enojärvi M, Armstrong D (1998). Analysis of oxidation and antioxidants using microtiter plates. Free radical and antioxidants protocols.

[63] Bitencourt GA, Chiari L, Valle CB, Laura VA, Moro JR (2011). Avaliação de diferentes métodos para extração de RNA total de folhas e raízes de braquiária. Embrapa—Boletim de Pesquisa e Desenvolvimento.

[64] Nair PMG, Chung IM (2014). A mechanistic study on the toxic effect of copper oxide nanoparticles in soybean (Glycine max L) root development and lignification of root cells. Biol Trace Element Res.

[65] Jassal PS, Kaur D, Prasad R, Singh J (2022). Green synthesis of titanium dioxide nanoparticles: development and applications. J Agricult Food Res.

[66] Hietzschold S (2019). Does nitrate reductase play a role in silver nanoparticle synthesis? Evidence of NADPH as the sole reducing agent. ACS Sustain Chem Eng.

[67] Javed R, Zia M, Naz S, Aisida SO, Ain NU, Ao Q (2020). Role of capping agents in the application of nanoparticles in biomedicine and environmental remediation: recent trends and future prospects. J Nanobiotechnol.

[68] Singh P, Garg A, Pandit S, Mokkapati VRSS, Mijakovic I (2018). Antimicrobial effects of biogenic nanoparticles. Nanomaterials.

[69] Neina, D. The role of soil ph in plant nutrition and soil remediation. Appl Environ Soil Sci 2019

[70] ALMEIDA, O. A. Qualidade da água de irrigação. Embrapa Mandioca e Fruticultura, Cruz das Almas. 2010. Disponível em: https://ainfo.cnptia.embrapa.br/digital/bitstream/item/26783/1/livro-qualidade-agua.pdf. Acesso em: 18 Nov. 2018.

[71] Miorini TJJ, Raetano CG, Everhart SE (2017). Control of white mold of dry bean and residual activity of fungicides applied by chemigation. Crop Protect.

[72] Choudhary K, Kataria J, Sharma S (2018). Evaluation of the kinetic and catalytic properties of biogenically synthesized silver nanoparticles. J Clean Prod.

[73] Zeilinger S, Gruber S, Bansal R, Mukherjee PK (2016). Secondary metabolism in Trichoderma—chemistry meets genomics. Fungal Biol Rev.

[74] Kubicek CP, Komon-Zelazowska M, Druzhinina IS (2008). Fungal genus Hypocrea/*Trichoderma*: from barcodes to biodiversity. J Zhejiang Univ Sci B.

[75] Vinale F (2009). Factors affecting the production of *Trichoderma* harzianum secondary metabolites during the interaction with different plant pathogens. LettAppl Microbiol.

[76] Verma M, Brar SK, Tyagi RD (2007). Antagonistic fungi *Trichoderma* spp panoply of biological control. Biochem Eng J.

[77] Troian RF, Steindorff AS, Ramadam H, Arrudaw CJU (2014). Mycoparasitism studies of *Trichoderma* harzianum against *Sclerotinia*
*sclerotiorum*: evaluation of antagonism and expression of cell wall-degrading enzymes genes. Biotechnol Lett.

[78] Haider AJ, Jameel ZN, Taha SY (2015). Synthesis and characterization of TiO2 nanoparticles via sol-gel method by pulse laser ablation. Eng Tech J.

[79] El-Desoky MM, Morad I, Wasfy MH, Mansour AF (2020). Synthesis, structural and electrical properties of PVA/TiO2 nanocomposite films with different TiO2 phases prepared by sol–gel technique. J Mater Sci Mater Electron.

[80] Jurić S, D̵ermić E, Topolovec-pintarić S, Bedek M, Vinceković M (2019). Physicochemical properties and release characteristics of calcium alginate microspheres loaded with trichoderma viride spores. J Integr Agric.

[81] EL-Moslamy SH, Elkady MF, Rezk AH, Abdel-Fattah YR (2017). Applying taguchi design and large-scale strategy for mycosynthesis of nano-silver from endophytic trichoderma harzianum SYA F4 and Its application against phytopathogens. Sci Rep.

[82] Lau ECHT, Carvalho LB, Pereira AES, Montanha GS, Corrêa CG, Carvalho HWP, Ganin AY, Fraceto LF, Yiu HHP (2020). Localization of coated iron oxide (Fe3O4) nanoparticles on tomato seeds and their effects on growth. ACS Appl Bio Mater.

[83] Park EJ (2008). Oxidative stress and apoptosis induced by titanium dioxidenanoparticles in cultured BEAS-2B cells. Toxicol Lett.

[84] Jaroenworaluck A (2006). Characteristics of silica-coated TiO2 and its UV absorption for sunscreen cosmetic applications. Surface Interface Anal.

[85] Weir A (2012). Titanium dioxide nanoparticles in food and personal care products. Environ Sci Technol.

[86] Schneider SL, Lim HW (2019). A review of inorganic UV filters zinc oxide and titanium dioxide. Photodermatol Photoimmunol Photomed.

[87] Dréno B, Alexis A, Chuberre B, Marinovich M (2019). Safety of titanium dioxide nanoparticles in cosmetics. J Eur Acad Dermatol Venereol.

[88] Fivenson D, Sabzevari N, Qiblawi S, Blitz J, Norton BB, Norton SA (2021). Sunscreens: UV filters to protect us: part 2-increasing awareness of UV filters and their potential toxicities to us and our environment. Int J Women's Dermatol.

[89] Morlando A (2018). Suppression of the photocatalytic activity of TiO2 nanoparticles encapsulated by chitosan through a spray-drying method with potential for use in sunblocking applications. Powder Technol.

[90] Benz D, Bui HV, Hintzen HT, Kreutzer MT, van Ommen JR (2022). Mechanistic insight into the improved photocatalytic degradation of dyes for an ultrathin coating of SiO2 on TiO2 (P25) nanoparticles. Chem Eng J Adv.

[91] Grande F, Tucci P (2016). Titanium dioxide nanoparticles: a risk for human health?. Mini-Rev Med Chem.

[92] Hamzeh M, Sunahara GI (2013). In vitro cytotoxicity and genotoxicity studies of titanium dioxide (TiO2) nanoparticles in Chinese hamster lung fibroblast cells. Toxicol In Vitro.

[93] Hanot-roy M (2016). Oxidative stress pathways involved in cytotoxicity and genotoxicity of titanium dioxide (TiO2) nanoparticles on cells constitutive of alveolo-capillary barrier in vitro. Toxicology Vitro.

[94] Bhattacharya K (2009). Titanium dioxide nanoparticles induce oxidative stress and DNA-adduct formation but not DNA-breakage in human lung cells. Particle Fibre Toxicol.

[95] Patel S, Patel P, Bakshi SR (2017). Titanium dioxide nanoparticles: an in vitro study of DNA binding, chromosome aberration assay, and comet assay. Cytotechnology.

[96] Armand L (2016). Long-term exposure of A549 cells to titanium dioxide nanoparticles induces DNA damage and sensitizes cells towards genotoxic agents. Nanotoxicology.

[97] Koca FD, Duman F (2018). Genotoxic and cytotoxic activity of green synthesized TiO2 nanoparticles. Appl Nanosci.

[98] Simonin M (2016). Titanium dioxide nanoparticles strongly impact soil microbial function by affecting archaeal nitrifiers. Sci Rep.

[99] Feizi H (2013). Phytotoxicity and stimulatory impacts of nanosized and bulk titanium dioxide on fennel (*Foeniculum* vulgare Mill). Chemosphere.

[100] Mahmoodzadeh H, Aghili R, Navabi M (2013). Physiological effects of TiO2 nanoparticles on wheat (*Triticum*
*aestivum*). Tech J Eng Appl Sci.

[101] Song U (2013). Functional analyses of nanoparticle toxicity: a comparative study of the effects of tio2 and ag on tomatoes (*Lycopersicon*
*esculentum*). Ecotoxicol Environ Safety.

[102] Antisari LV (2015). Uptake and translocation of metals and nutrients in tomato grown in soil polluted with metal oxide (Ceo, Fe3O4, SnO2, TiO2) or metallic (Ag Co, Ni) engineered nanoparticles. Environ Sci Poll Res.

[103] Song G (2012). Physiological effect of anatase TiO2 nanoparticles on *Lemna* minor. Environ Toxicol Chem.

[104] Mahmoodzadeh H, Navabi M, Kashefi H (2013). Effect of nanoscale titanium dioxide nanoparticles on the germination and growth of canola (*Brassica*
*napus*). J Ornam Horticult Plants.

[105] Khot LR (2012). Applications of nanomaterials in agricultural production and crop protection. Crop Prot.

[106] Foltête AS (2011). Environmental impact of sunscreen nanomaterials: ecotoxicity and genotoxicity of altered TiO2 nanocomposites on Vicia faba. Environ Poll.

[107] Pandey V, Awasthi M, Singh S, Tiwari S, DWIVEDI U (2017). A comprehensive review on function and application of plant peroxidases. Biochem Anal Biochem.

[108] Cunha-Lopes TL, Siqueira-Soares RC, Almeida GHG, Melo GSR, Barreto GE, Oliveira DM, Santos WD, Ferrarese-Filho O, Marchiosi R (2018). Lignin-induced growth inhibition in soybean exposed to iron oxide nanoparticles. Chemosphere.

[109] Cerny M, Habánová H, Berka M, Luklová M, Brzobohatý B (2018). Hydrogen peroxide: its role in plant biology and crosstalk with signalling networks. Int J Mol Sci.

